# Device-measured movement behaviours and cancer incidence in a population sample of UK adults: Dual 24-hour analyses of postural and intensity compositions

**DOI:** 10.1186/s12916-026-05069-3

**Published:** 2026-07-18

**Authors:** John J Mitchell, Raaj Kishore Biswas, Nicholas A. Koemel, Matthew N. Ahmadi, Joanna M Blodgett, Karen Canfell, I-Min Lee, Christine M Friedenreich, Armando Teixeira-Pinto, Peter A. Cistulli, Dorothea Dumuid, Anthony D. Okely, Abigail Fisher, Mark Hamer, Emmanuel Stamatakis

**Affiliations:** 1 Institute of Sport Exercise & Health, Division of Surgery and Interventional Sciences, Faculty of Medical Sciences, University College London, London, UK; 2 Charles Perkins Centre, Faculty of Medicine and Health, University of Sydney, Sydney, NSW Australia; 3 Turner Institute for Brain and Mental Health, School of Psychological Sciences, Monash University, Melbourne, VIC, Australia; 4 Mackenzie Wearables Research Hub, Faculty of Medicine and Health, University of Sydney, Sydney, NSW Australia; 5 Victorian Heart Institute, Monash, Melbourne, Australia; 6 National Institute for Health and Care Research Biomedical Research Centre, University College London Hospitals, London , UK; 7 The Daffodil Centre, University of Sydney and Cancer Council NSW, Sydney, NSW Australia; 8 Department of Medicine, Harvard Medical School, Boston, MA 02115 USA; 9 Division of Preventive Medicine, Department of Medicine, Brigham and Women’s Hospital, Harvard Medical School, Boston, MA 02215 USA; 10 Department of Epidemiology, Harvard T. H. Chan School of Public Health, Harvard University, Boston, MA 02115 USA; 11 Departments of Oncology and Community Health Sciences, Cumming School of Medicine, University of Calgary, Calgary, AB Canada; 12 Cancer Care Alberta, Calgary, Canada; 13 Centre for Kidney Research, Kids Research Institute, The Children’s Hospital, Westmead, NSW Australia; 14 Sydney School of Public Health, University of Sydney, NSW Sydney, Australia; 15 Alliance for Research in Exercise, Nutrition and Activity (ARENA), Allied Health and Human Performance, University of South Australia, Adelaide, 5000 Australia; 16 Centre for Adolescent Health, Murdoch Children’s Research Institute, Parkville, 3052 Australia; 17 Australia and Early Start, Faculty of the Arts, Social Sciences and Humanities, University of Wollongong, Wollongong, Australia; 18 School of Health and Society, Faculty of the Arts, Social Sciences and Humanities, University of Wollongong, Wollongong, Australia; 19 Research Department of Behavioural Science and Health, University College London, London, UK

**Keywords:** Posture, Wearables, Accelerometry, Movement behaviour, Cohort studies, Epidemiology, Machine learning

## Abstract

**Background:**

Insufficient physical activity (PA) and excessive sedentary behaviour is associated with several cancers. Personalised approaches to increasing healthy movement behaviours over unhealthy behaviours may be more effective than a one-size-fits-all approach. Exploring both postures, and intensities across the 24-hour day may reveal actionable behavioural alternatives from current guidance.

**Methods:**

Using a novel dual-compositional approach, we assessed how differences in participant’s composition of 24-hour daily movement (postures and intensities) and sleep are differentially associated with PA-related cancer incidence (a composite of 13 sites linked with physical inactivity). This prospective analysis involved adults drawn from the UK Biobank accelerometry subsample each followed-up by health linkage. Participant’s daily movement was classified into two 24-hour compositions. Composition 1 (posture-focused): sleep duration, sedentary behaviour (SB), standing, moving at any intensity. Composition 2 (intensity-focused): sleep duration, sedentary time (ST), light PA (LPA), moderate PA (MPA), and vigorous PA (VPA). Secondary analysis combined VPA and MPA as moderate-to-vigorous PA (MVPA). PA-related cancer diagnoses were captured from health registry data for up to 9.5 years (y). Cox-proportional hazards models were adjusted for age, sex, education, smoking, alcohol, diet, parental cancer history, cardiovascular disease and medication use.

**Results:**

Analyses included 59,218 (55% female) participants (mean [SD] age: 61.7 [7.8]y), with a median follow-up of 8.0y [IQR: 7.4-8.5y; 464,640 person years] with 2,385 (4%) incident cancer events. Among the average, active participant, greater moving in place of other behaviours was associated with lower cancer risk, e.g. theoretically replacing 15 min of sleep or SB with 15 min of moving was associated with hazard ratios (HR) of 0.98 (95% Confidence Interval (95%CI): 0.97–0.99) and 0.98 (95%CI: 0.97–0.99), respectively. Similar risk reduction was observed with 30 min additional standing in place of sleep or SB. Regarding intensity, greater MVPA, in place of any behaviour proved most robustly associated with lower risk, although notably, the VPA component within MVPA proved the critical intensity.

**Conclusions:**

Beyond MVPA, moving at any intensity, in place of other postures, was associated with reduced risk of cancer. However, greater standing may also provide a plausible behavioural adjunct or alternative, warranting further investigation.

**Supplementary Information:**

The online version contains supplementary material available at 10.1186/s12916-026-05069-3.

## Background

Cancer is a leading cause of mortality globally [1, 2]. In 2022, there were an estimated 20 million new cancer diagnoses and 9.7 million deaths attributed to cancer [3]. Recent consensus statements on cancer prevention have focussed on lifestyle behaviours, finding physical inactivity among the most modifiable of risk factors and a target for intervention [4]. Regular engagement in leisure-time physical activity (LTPA) has been previously associated with an 8–25% reduction in cancer risk across a wide range of cancer sites including upper gastrointestinal and liver cancers, and even highly prevalent cancers, including lung, breast and colorectal cancers [4, 5]. Nevertheless, physical activity (PA) is only one facet of the 24-hour (h) daily movement spectrum, alongside sleep and sedentary behaviour (SB) [6], and also encompasses a spectrum of postures and movement intensities. Given the finite nature of time use, it is crucial for public health guidance to understand which behaviours should be a focus of behaviour change interventions for cancer risk reduction. Studies have also reported an adverse relationship between SB, and cancer, independent of PA [7–10], while the literature focusing on sleep is largely inconclusive [11]. Despite these independent lines of evidence, the field has increasingly acknowledged the interplay between all daily movement behaviours, including SB and sleep for health and longevity [12–14].

The World Health Organization (WHO) recommends 150–300 min of moderate-intensity PA, or 75 min of vigorous-intensity PA, weekly [15, 16]. There is however. a growing interest in 24-h guidelines for movement behaviour, which encompass PA, SB and sleep, each of which have health consequences, and therefore multiple possible behaviour change options may yield health benefits [13, 17]. The 24-h movement continuum may be characterised according to both posture and movement intensity, and can be studied through 24-h accelerometery [18–22]. When combined with compositional data analysis, which can model all movement in the 24-h day simultaneously, while focusing on the sample average day, it is possible to estimate the difference in risk associated with even modest differences in average daily movement [23].

Despite their methodologic strengths, prior studies that have used a compositional approach typically examine only one dimension of movement behaviour (intensity), resulting in a narrow range of recommendations [12, 24]. Given the high-proportion of individuals globally who remain physically inactive [25], and the need to accommodate for the needs of those for whom find it challenging to meet the intensity-based guidelines, we sought to explore multiple dimensions of movement behaviour. Each of these dimensions (e.g. posture/intensity) may be clinically useful and adaptable to different circumstances. Accordingly, we adopted a dual-approach; separately modelling two compositions of movement behaviour, sedentary time and sleep in relation to cancer risk.

Using a comprehensive dual 24-h compositional approach, this study examines both postures (SB, standing, moving) which so far have received little attention in relation to cancer risk, and intensities (sedentary time (ST), and light, moderate and vigorous intensity PA), and sleep in relation to cancer risk.

## Methods

### Study population

Participants were drawn from the UK Biobank Study, a prospective cohort study involving 502,629 individuals within the UK, aged 40–69 years (y) at the time of enrolment in 2006 to 2010. Participants provided informed consent before undertaking a range of physical examinations by trained practitioners and completing health and lifestyle questionnaires.

### Measurement of posture, physical activity, and sleep

The UK Biobank (UKBB) wrist accelerometry sub-study [26], which received ethical approval from the UK National Research Ethics Service (No. 11/NW/0382), recruited 103,684 individuals from June 2013 to 2015. Sample derivation is displayed in Additional file 1: Fig S1. Participants wore an Axivity AX3 accelerometer device (Axivity, Newcastle, UK) on their dominant wrist for 7 days [26]. Devices were initialised to collect movement data with a sampling frequency of 100 Hz and a dynamic range between ±8 g. A valid day encompassed a minimum of 16 h (h) of wear time [27]. Participants were included if they accrued a minimum of three days valid wear, including at least one weekend day. Monitors were calibrated [28], corrected for orientation [29], and non-wear detected using standard procedures in UKBB [27, 30]. The sleep duration algorithm was developed and validated in 3,752 British participants with sleep diary data and 28 participants with polysomnography data [31] (further details provided in Additional file 1: Text S1).

Two further activity and posture classifiers, validated in laboratory and free-living conditions [32, 33] enabled waking time to be characterised into compositions of intensity, and postures.

To study both posture and intensity, we utilised a novel dual-composition approach [34]. The two compositions were defined as follows:

### Composition 1: Posture (4-parts)

The activity classification scheme uses features from raw acceleration signals to classify waking-periods as sitting, standing, or walking/running in 60-second (s) windows [32, 33], using a Random-forest classifier with a Hidden Markov Model [35]. Under free-living conditions, the classifier achieved balanced accuracy (average of sensitivity and specificity) of 88% for SB and 80% for standing [33]. Composition 1 comprised: sleeping, SB, standing, and moving (Additional file 1: Text S1) [18, 27, 31–33].

### Composition 2: Intensity (5-parts)

Physical activity intensity was classified with a previously adopted two-level Random Forest algorithm [18, 27]. The first level classifies activity type (moving activities, standing utilitarian movements (semi-stationary) and sedentary time, in contiguous 10s bins. The second level further categorizes activities into intensity bands. Moving activities are grouped by their acceleration into light PA, moderate PA, and vigorous PA. The intensity composition encompassed five behaviours: sleep, ST, light PA (LPA), moderate PA (MPA) and vigorous PA (VPA) in minutes (min). A secondary intensity (4-part) composition combined MPA and VPA to MVPA.

For both compositions, the average daily time in each component across the week was normalised to 24 h [36] (average wear-time across valid days was 23 h 26 min), and a log-ratio expectation maximisation algorithm was utilised to replace rounded zeroes [37, 38].

### Physical activity-related cancer incidence

We used a single composite outcome comprising incidence of cancer at any one of 13-sites shown to be associated with leisure-time PA [5, 39]. This PA-related cancer composite variable encompassed bladder, colon, endometrial, gastric cardia, head and neck, kidney, liver, breast, lung, rectal cancer, myeloma, oesophageal adenocarcinoma and myeloid leukaemia (Additional file 1: Table S1). Incidence and date of diagnosis was derived via linkage with the National Health Service (NHS) Digital records for participants based in England and Wales, while the NHS Central Register was used to identify cancer events in participants in Scotland. Inpatient hospitalisation data were provided by either the Hospital Episode Statistics for England, the Patient Episode Database for Wales, or the Scottish Morbidity Record for Scotland. For England and Wales, cancer diagnosis data were followed-up through to 31 December 2020 and 31 December 2016 respectively [40], and for Scotland, diagnosis data were followed-up through 30 November 2021. Deaths during follow-up, including from non-PA-related cancers were derived from the same registries. Differences in censoring dates arise due to the availability and timing of linked national cancer registry and hospital data updates across UK nations. Follow-up commenced from the date of accelerometer assessment (02/2013-12/2015) until incident cancer diagnosis, death, loss to follow-up, or administrative censoring. Participants with any history of cancer (*n* = 7,633) were excluded.

### Covariates

Covariates were selected a priori based on previous literature [5, 39]. Covariates included age (years), sex at birth (male vs. female), ethnicity (white vs. any other ethnic background) and maximal educational level (College/University (attained at > 18y; A/AS level (attained at 18y); O levels (attained at 16y); NVQ/HND/HNC (typically attained at > 16y); CSE (attained at 16y); other). Health factors included cardiovascular disease (CVD), captured by self-report or hospital admission (yes/no), medication use (taking antihypertensives, anti-hypercholesterolaemia or insulin) and family history of cancer (yes/no). Lifestyle factors included smoking status (never, past, current), diet quality (daily fruit/vegetable servings) alcohol consumption history (never, ex, current within UK guideline levels (< 15 weekly units), current above guidelines (≥ 15 weekly units)). Further derivation details are provided in Additional file 1: Table S1. Adiposity measures were not included in the fully adjusted models due to their hypothesised mediating role between 24-h movement behaviour and cancer [21]. However, a sensitivity model was fitted, adjusting for body mass index (BMI, kg/m^2^).

### Analyses

Primary analyses involved an established isometric-log ratio (ILR)-coordinate compositional approach [23, 36, 41, 42] using Cox proportional hazards models. This statistical method examines the association of each behaviour, relative to all others, with the outcome. Further details on the modelling procedure are provided in Additional file 1: Text S2 [41, 43]. Fine-Gray sub-distribution method accounted for the competing risk of mortality from all-causes including non-PA-related cancer during the follow-up window. Hazard ratios therefore reflect subdistribution hazard ratios, reflecting cumulative cancer incidence in the presence of competing mortality. The proportional hazards assumption was examined by inspecting Schoenfeld residuals for each model, revealing no violations. The first analyses studied the 4-part composition defined by posture; the second studied the 5-part composition defined by intensity. Each component was examined relative to all others in the composition, in separate models until all possible combinations had been studied. For clinical relevance, secondary analysis repeated the primary models with the four-part intensity composition involving MVPA in place of MPA and VPA and also using a VPA:MPA ratio-variable to examine their relative importance.

Minute-by-minute isotemporal substitution then assessed the difference in absolute risk from theoretically reallocating time from those behaviours significantly associated with cancer, into/in place of each other behaviour, whilst holding all others fixed at the sample average. These theoretical reallocations represent risk differences between-individuals and not within-individual change. To provide insights relevant to future interventions [39, 44], estimated change in risk was reported for reallocation increments of 3,5,10,15,30,45,60 and 90 min (selected to be informative for the full range of behaviours, while modelling within the limits of the data).

### Sensitivity analyses

Participants with cancer events occurring in the first and second year of follow-up were sequentially removed, to assess the possibility of reverse causation [39]. E-values were then calculated to estimate the risk of residual confounding [45]. Models were also fitted stratified by cancer site. Finally, we compared the analytical and excluded samples to assess possible selection bias.

## Results

### Participant demographics

A total of 59,218 participants (55% female) were included (mean age in years (standard deviation): 61 (7.8)) at baseline (Additional file 1: Fig. S1). A total of 2,385 (incidence rate: 4.0%) new PA-related cancer events occurred in the follow-up window (median follow up: (interquartile range (IQR)): 8.0 [7–9] years; table S2) and 1,552 participants died during follow-up (Table S3).

The included sample were predominantly of white ethnic backgrounds (96%). A large proportion had a college or university degree (43%), never smoked (56%) and consumed alcohol within guideline levels (57%) (Table 1).

**Table 1:** Included (*n* = 59,218) UK Biobank participant characteristics at baseline accelerometry collection (June 2013–2015)

		Total Sample	
		*n* = 59,218	
**Age (years)**	Mean, SD	61.7	7.8
**Sex (female)**	n, %	32,452	55%
**Ethnicity (White)**	n, %	56,988	96%
**Education**	n, %		
College/University degree		25,737	43%
A/AS-level		7966	13%
NVQ/HND/HNC		3235	6%
CSE		2335	4%
O-levels		12,116	21%
Other		7829	13%
**Smoking history**	n, %		
Current		3925	7%
Never		34,092	56%
Ex-smoker		21,201	37%
**Medication Use** (Cholesterol lowering, Blood pressure-lowering, Insulin)	n, %	13,549	23%
**Alcohol**	n, %		
Above guidelines		22,409	38%
Ex-alcohol drinker		1545	3%
Within guidelines		33,647	57%
Never		1617	3%
**Family History of Cancer**		15,353	26%
**Diet Quality (Fruit/Vegetable consumption) index**	Median (Q1,Q3)	7	(5–10)
**Previous History of CVD**		5366	9%
**PA-related Cancer Incidence**		2385	4%
**Follow up in years**	Median (Q1,Q3)	8	(7.4–8.5)

Abbreviations: CVD: Cardiovascular disease; PA: Physical activity; NVQ: National Vocational Qualification; HND: Higher National Diploma; HNC: Higher National Certificate; CSE: Certificate of Secondary Education. Eligible sample included complete Physical activity measures, cancer outcomes and/or covariates (excluded sample presented in Additional file 1: Table S15)

### Participant movement compositions

**Fig. 1:**
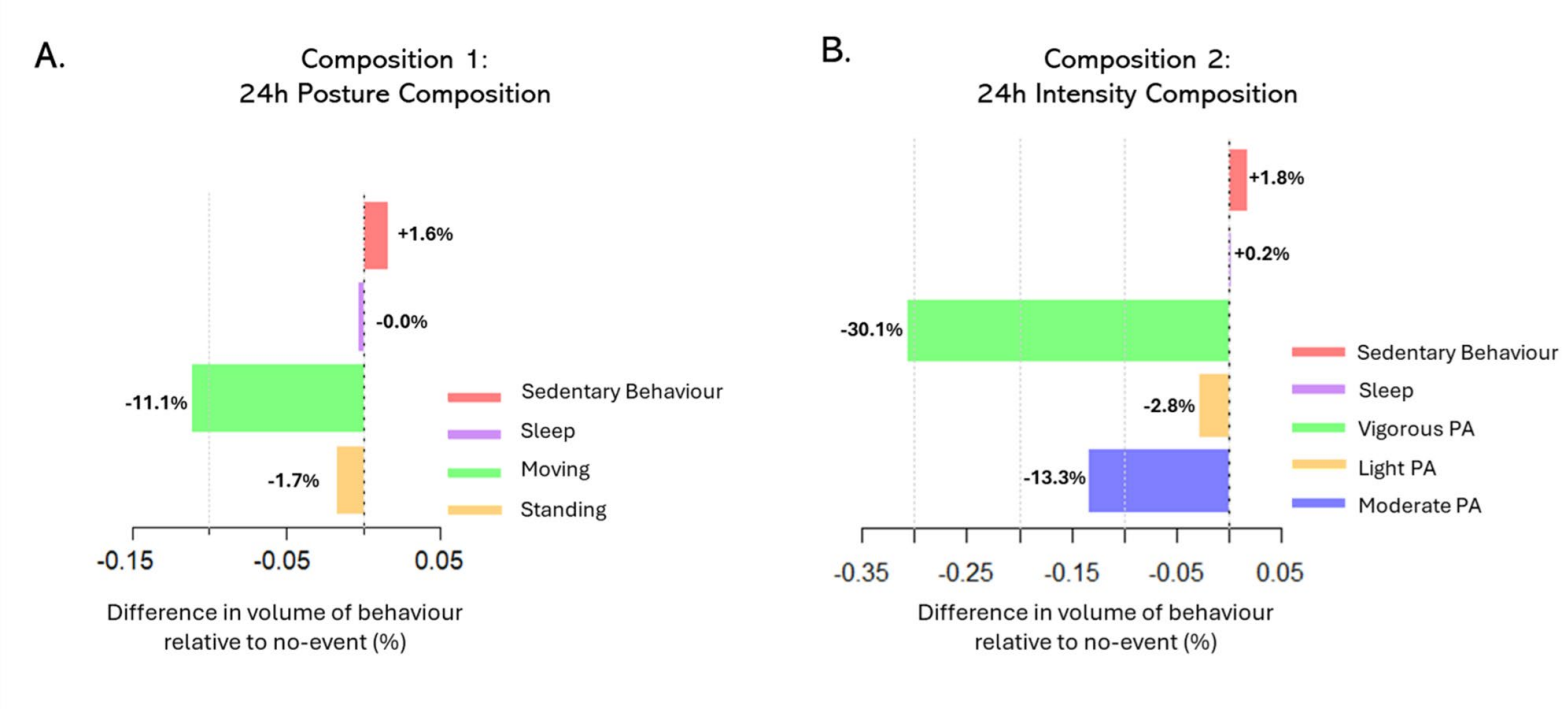
Proportional differences in movement (posture and intensity) and sleep compositions between participants with physical activity-related cancer vs. no cancer. Proportional differences in the compositional average of movement behaviour, grouped by **A**. posture and **B**. intensity, of participants with incident PA-related cancer event in the follow-up window (n=2,385) relative to participants with no PA-related cancer event (n=55,281) over a median follow-up of 8.2 years

The overall sample mean for composition 1 (posture) was 7 h 39 min sleeping, 11 h 13 min of SB, 4 h 17 min standing, and 51 min moving (Additional file 1: Fig. S2A). Participants who subsequently developed cancer accrued less time moving, and standing, and greater SB at baseline compared with those without a cancer diagnosis during the follow-up period. There were no clear differences in sleep duration (Fig. 1A).

The overall sample mean for composition 2 (intensity) was 7 h 42 min sleeping,10 h 44 min of ST, 5 h 3 min of LPA, 28 min of MPA, and 3 min of VPA (Additional file 1: Fig. S2B). Individuals with a subsequent cancer diagnosis spent less time in VPA, MPA and LPA and more time sedentary at baseline than those with no subsequent diagnosis (Fig. 1B).

### Associations between Composition 1 (24-h posture) and cancer incidence

**Fig. 2:**
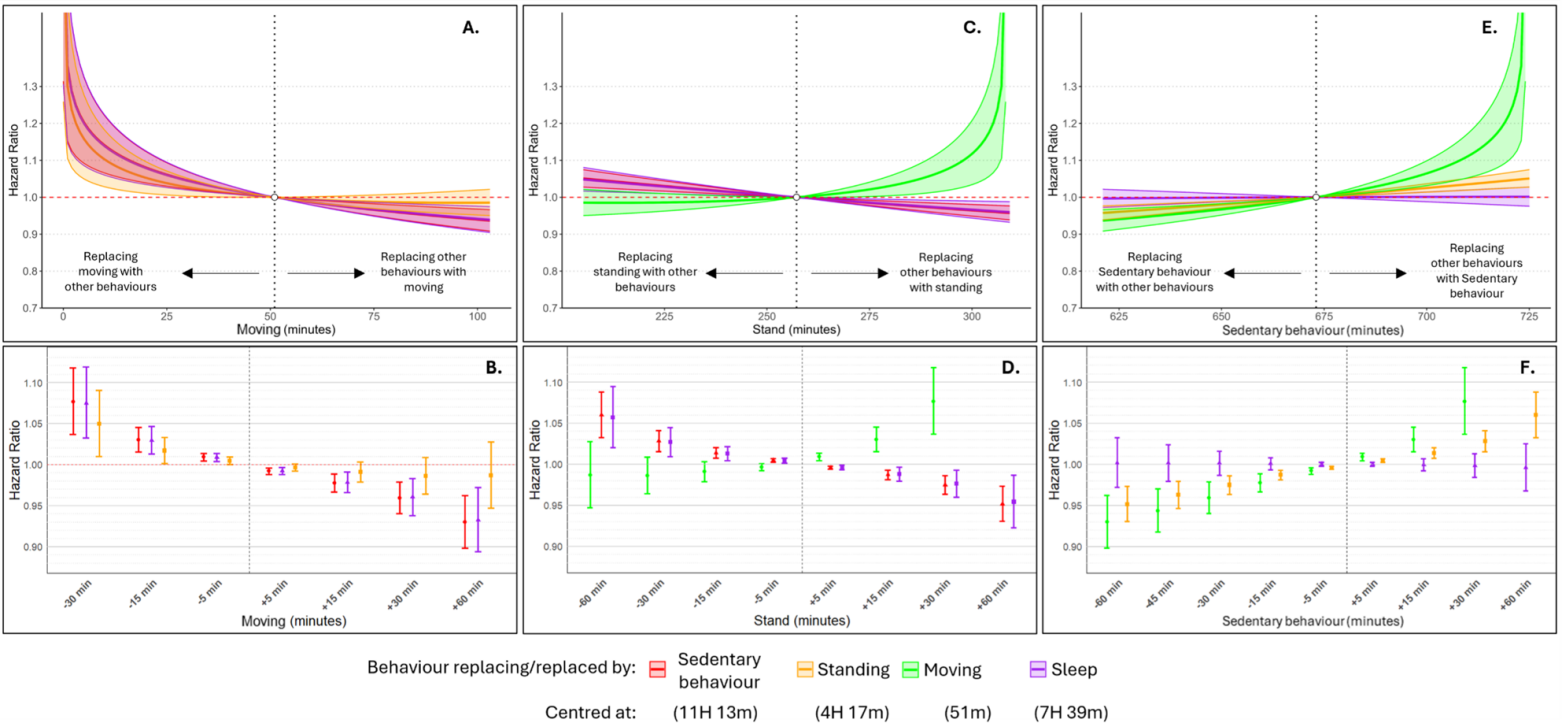
Difference in physical activity-related cancer hazard from theoretical reallocation of time between composition 1 (postures). Estimated difference in physical activity (PA)-related cancer hazard from theoretical reallocation of moving (**A**,** B**) in hours (H)/minutes (m), standing (**C**,** D**) and sedentary behaviour (**E**,** F**) with/in place of each other postures and sleep, while holding each other behaviour constant around the sample average. Isotemporal substitution is performed on compositional Fine-Gray cox proportional hazards model, with 2,385 PA-related cancer events in *n* = 59, 218; median 8.2y follow-up, and adjusted for age, sex, ethnicity, smoking status, alcohol use, diet quality, family history of cancer, history or presence of cardiovascular disease and regular medication use. Omitted bars are theoretical substitutions which exceed the range of the compositional data

A greater proportion of time spent moving or standing, each relative to all other behaviours, was inversely associated with cancer incidence, while greater SB, relative to other behaviours was associated with greater cancer risk (Table S4). The greatest difference in cancer risk from theoretical behavioural changes to the sample average Composition 1 involved replacing sleep or SB with moving (Fig. 2). For example, replacing 15 min of sleep or 15 min of ST with moving were each associated with HR of 0.98 (95% Confidence Interval: 0.97–0.99; Fig. 2A-B, Additional file 1: Tables S5-S7). However, replacing sleep or SB with standing required upwards of 30 min of replacement for equivalent risk reduction (30 min sleep replaced with 30 min standing, HR: 0.97, 95%CI: 0.96–0.99; 30 min SB replaced with 30 min Standing, HR: 0.98, 95%CI: 0.96–0.99; Fig. 2C-D).

Conversely, compositions with less standing or moving and greater SB or sleep were associated with a greater cancer incidence (30 min moving replaced with 30 min SB, HR: 1.08, 95%CI: 1.04–1.12; 30 min moving replaced with 30 min sleep, HR: 1.07, 95%CI: 1.03–1.12; 30 min standing replaced with 30 min SB, HR: 1.03, 95%CI: 1.02–1.04; 30 min standing replaced with 30 min sleep, HR: 1.03, 95%CI: 1.01–1.04; Fig. 2E-F).

### Associations between Composition 2 (24-h movement intensity) with cancer incidence

**Fig. 3:**
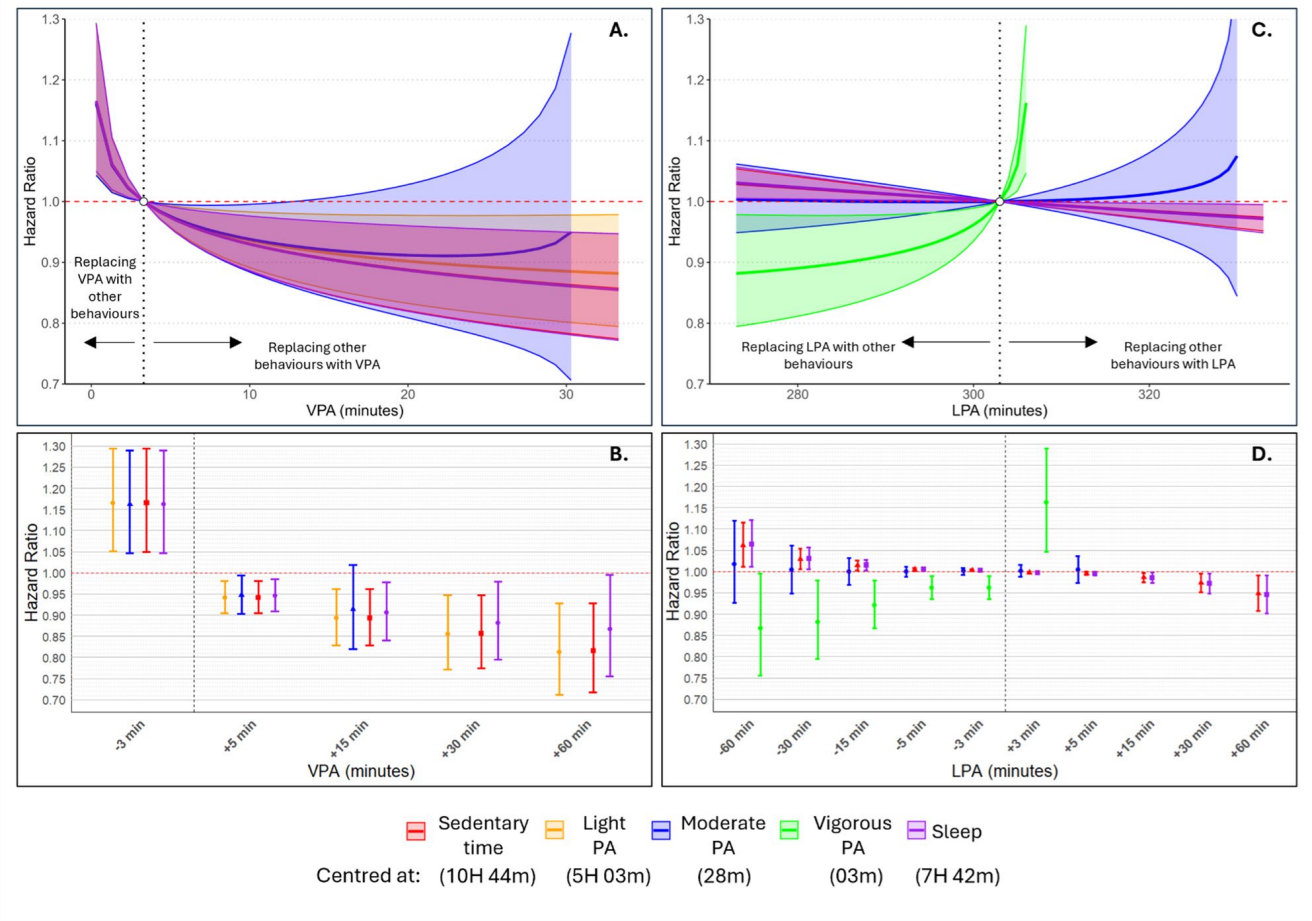
Difference in physical activity-related cancer hazard from theoretical reallocation of time between composition 2 (intensity). Estimated difference in physical-activity (PA)-related cancer hazard from theoretical reallocation of vigorous physical activity (VPA) (**A**,** B**) in hours (H)/minutes (m) and light physical activity (LPA) (**C**,** D**) into/in place of each other movement intensity and sleep, while holding each other behaviour constant around the sample average. Isotemporal substitution is performed on compositional Fine-Gray cox proportional hazards model, with 2,385 PA-related cancer events in *n* = 59, 218; median 8.2y follow-up, and adjusted for age, sex, ethnicity, smoking status, family history of cancer, history or presence of cardiovascular disease, alcohol, diet quality, and regular medication use. Omitted bars are theoretical substitutions which exceed the range of the compositional data

Greater time in VPA and LPA, each relative to all other behaviours, was inversely associated with cancer incidence (Table S8). Theoretically increasing daily VPA in place of other behaviours was associated with the greatest cancer risk reduction (Fig. 3, Additional file 1: Tables S9,S10). Only 3 min additional VPA in place of any other intensity was associated with lower cancer risk (3 min sleep replaced with 3 min VPA, HR: 0.96, 95%CI: 0.93–0.99; 3 min ST replaced with 3 min VPA, HR: 0.96, 95%CI: 0.93–0.99; 3 min LPA replaced with 3 min VPA, HR: 0.96, 95%CI: 0.93–0.99; 3 min MPA replaced with 3 min VPA, HR: 0.96, 95%CI: 0.93–0.99). Comparable risk reduction from LPA required in excess of 90 min, in place of sleep (HR: 0.92 (0.86–0.99)) or ST (HR: 0.92 (0.87–0.99)).

Similar to composition 1 (postures) reallocating time from VPA or LPA into ST or sleep was associated with increased cancer risk (Fig. 3A-D).

When re-fitted with MVPA, greater time in MVPA, relative to other behaviours was inversely associated with cancer risk (Table S11). However, cancer risk declined as the proportion of VPA within MVPA increased (Additional file 1: Fig. S3).

### Sensitivity analysis

E-values indicated that an unmeasured confounder must have an association of HR:1.12–1.24, and 1.19–1.63 with both the exposure and outcome to nullify the weakest, and strongest reported associations respectively (Additional file 1: Tables S12, S13). Removing the first (*n* = 243 events) and second year (*n* = 533 events) of follow-up did not alter the results (Additional file 1: Tables S14-S17), however, associations between cancer risk and VPA were no longer significant after removing the first two years of events, and average VPA was marginally (< 1 min/day) lower amongst these individuals, despite minimal differences in their overall compositions (Figures S4-5). Models involving MVPA proved robust in all samples (Table S18). When subdivided by cancer site, the pattern of associations proved directionally consistent as with the cancer composite outcome. Subtle differences included breast cancer, which was consistently null, despite being the site with highest prevalence (Figures S6,S7). Excluded individuals (first 2y of events) were older and proportionally more female (Table S19). Finally, adjustment for BMI fully-attenuated the association between SB, relative to all other postures, with the outcome.

## Discussion

We adopted compositional data analysis methods to model the full spectrum of 24-h movement behaviours (categorised by posture and intensity) in relation to cancer incidence. When examining the posture composition, more time (15 min) moving or more time standing above the average was associated with ~ 1–7% lower cancer risk, while greater time spent sedentary was associated with greater risk. When categorised by intensity, greater MVPA (particularly when comprising substantial VPA) relative to other behaviours, followed by greater LPA relative to other behaviours, was associated with lower cancer risk. As in the posture composition, greater time spent sedentary was associated with exponentially greater risk. These findings may provide the basis for a broader range of options for recommendations for 24-h movement behaviour for mitigating cancer risk, enhancing applicability to the wider population.

Few large-scale studies have investigated the role of daily movement, including SB and sleep with risk of cancer [46, 47]. The extant literature has predominantly relied on self-reported PA [46] and SB [7, 8, 46] and separately implicates leisure-time PA as protective, and SB as harmful [5, 10, 46, 48]. One device-based study extends this finding to daily behaviour, demonstrating the major potential of even short bursts of VPA accrued throughout the day for reducing cancer risk [39]. Stepping too has been linked to lower cancer risk revealing an inverse dose-response association up until 5–7,000 steps/day [49]. Finally, one device-based study has formally applied a 24-h movement framework in relation to cancer risk, revealing greater MVPA in the 24-h day to be most inversely associated with cancer incidence [24]. Our study aligns with findings from device-based studies by (i) underscoring the potential benefits of moving postures, and particularly MVPA [24] and (ii) harms of excessive sitting [10]. However, it extends findings by (iii) revealing the importance of VPA when combined with MPA, and (iv) applies a dual-compositional approach to reveal, for the first time to our knowledge, how reallocating time to standing from sitting or sleep, may provide a potential protective benefit. This study thus provides the most detailed and nuanced exploration of 24-h movement behaviours and cancer risk to-date. Site specific analyses were broadly consistent with prior evidence [24], but with the notable difference for breast cancer which did not show evidence of associations within our study, despite its high prevalence. These findings must therefore be interpreted cautiously as associations did show differences by specific cancer-site [24].

There are several hypothesized biologic mechanisms that may explain these findings. Some evidence exists that physical activity reduces chronic inflammation and insulin resistance while SB may increase these metabolic imbalances [50, 51]. Atypically, the less consistent finding for MPA alone may suggest that its combination with more intense movement is needed to trigger the physiological mechanisms that are critically related to cancer pathways [51]. Although evidence of this phenomenon specific to cancer remains limited, similar intensity-dependent patterns have been reported for other chronic disease outcomes, particularly CVD, where the proportion of vigorous activity accumulated within a combined MVPA intensity variable appears to confer additional benefit beyond total activity volume alone. This suggests that VPA and MPA are not physiologically equivalent, nor eliciting identical pathways, but instead that their combination is more potent than either individual intensity alone [52]. LPA however, which is performed in larger volumes than MPA or VPA, may act through different mechanisms for cancer risk reduction, such as contributing more to overall daily movement and energy expenditure by minimising SB, which in all analyses appeared theoretically harmful. These proposed mechanisms are further supported by our finding that adjustment for BMI nullified the associations for SB, further suggesting adiposity may heavily mediate associations between SB and cancer. When grouped by posture, the potential mechanisms—in particular mechanisms linking standing with cancer—remain less clear [53]. We hypothesise that standing postures, as with LPA, may primarily act to break-up SB bouts which increase cancer risk [7, 22]. The observed lack of associations of sleep with cancer align with the largest meta-analysis on this topic, which found limited evidence of an association with cancer for either long or short self-reported sleep duration [11]. Although beyond the scope of our study, it is possible that other dimensions of sleep (including quality and consistency) and the co-existence of sleep disorders (e.g. sleep apnoea), may still be mechanistically more important for shaping cancer risk, beyond duration alone [54].

The above findings have substantial implications for public health policy. Where current movement guidelines focus exclusively on intensity and adopt a one-size-fits-all approach, we show how both changes in postural and intensity (e.g. increasing positive postures and more intensive movement), alongside reducing SB may act synergistically. However, some behavioural differences may be more realistic and therefore actionable in different contexts than others. For example, risk differences for compositions with greater LPA in place of other behaviours required > 90 additional min in place of ST, which may be less feasible than attaining 3–5 additional min of VPA as part of their daily MVPA. Further to these findings, and the hypothesis-generating findings for posture, future research is needed which examines these associations in different contexts (e.g. amongst non-exercisers and those at the extreme minimum levels of activity) to confirm if these findings could be the basis for personalised behaviour change interventions for effective cancer prevention. Critically, further investigation is needed to understand the nuance of these associations by individual cancer site, given the observed differences in strength of associations by site.

### Strengths and limitations

Strengths include the unique dual composition approach combined with contemporary approaches for the measurement, processing of 24-h movement data from accelerometers. Validated in free-living conditions, these procedures greatly enhanced the ability to capture dimensions of movement -posture and intensity– largely impossible using self-reported measures. Specifically, the use of compositional analysis permits the examination of all movement behaviours in tandem; a major limitation of prior studies that examine one or few behaviours, independently. Secondly, this study provides informative metrics on optimal dose to guide intervention efforts. Finally, procedures were used to account for multiple possible sources of bias, including reverse causation and quantification of residual confounding.

There were limitations. Most notably, the isotemporal substitution analyses represent between-individual differences in PA-related cancer risk, associated with differences in 24-h composition. Interpreting the theoretical reallocations therefore requires caution, as each cancer site often has complex aetiologies which develop over many months, years or decades and the favourable effects of behavioural interventions likely require similarly extensive or chronic periods to observe measurable reduction in risk. The adopted ILR approach also does not typically include non-linear terms which may be relevant for modelling certain behaviours. Further, the small E-values leave open the possibility for residual confounding nullifying findings smallest in magnitude. The focus on the chosen cancer sites was based on self-reported measures of LTPA [5], of which there is incomplete consensus [46], and other cancer types may still show associations with other facets of daily movement, including sleep. The direction of these associations, in particular for standing and light activity, may also differ by domain (e.g., occupational versus leisure-time) which cannot be captured with accelerometry alone [55]. The UK Biobank study has known healthy-volunteer bias, and has a low proportion of individuals from non-white ethnic backgrounds whom may have site-specific differences in cancer risk [56]. This reduces generalisability of the findings. Further, bias may arise from reactivity to health behaviour monitoring during the accelerometer wear period [57]. However, previous empirical evidence suggests that the poor representativeness of the UK Biobank did not materially affect the associations of PA with cancer outcomes [58]. Finally, use of a global adjustment set may also not fully account for the nuanced confounding pathways which are specific to each site.

## Conclusions

This study finds greater time spent either (i) standing or moving in place of SB and sleep, (ii) attaining MVPA in place of any behaviours, particularly when comprising substantial VPA, or (iii) attaining LPA in place of sleep or ST are associated with lower cancer risk, providing a wider range of options for movement behavioural change to be explored by future interventions.

## Supplementary Information

Below is the link to the electronic supplementary material.

Supplementary Material 1: Supplementary material, Tables S1-S19, Text S1-S2 and Fig. S1-S7

## Data Availability

The data that support the findings of this study are available from theUK Biobank but restrictions apply to the availability of these data, which were used underlicense for the current study, and so are not publicly available.

## Abbreviations

PA: Physical activity
VPA: Vigorous physical activity
LPA: Light physical activity
MPA: Moderate physical activity
SB: Sedentary behaviour
ST: Sedentary time
h: Hour
min: Minute
WHO: World Health Organization
CVD: cardiovascular disease
