# Supplementary material for: Device-measured movement behaviours and cancer incidence in a population sample of UK adults: Dual 24-hour analyses of postural and intensity compositions
Source: BMC Med. 2026 Jul 18;24:427. doi: 10.1186/s12916-026-05069-3 (PMC13455226; doi:10.1186/s12916-026-05069-3)
Supplement: Supplementary file 1 [file 12916_2026_5069_MOESM1_ESM.pdf]

Additional file 1 (Supplementary material): Device-measured movement behaviours and cancer incidence in a population sample of UK adults: Dual 24-hour analyses of intensity and postural compositions

Figure S1. Sample derivation flow chart

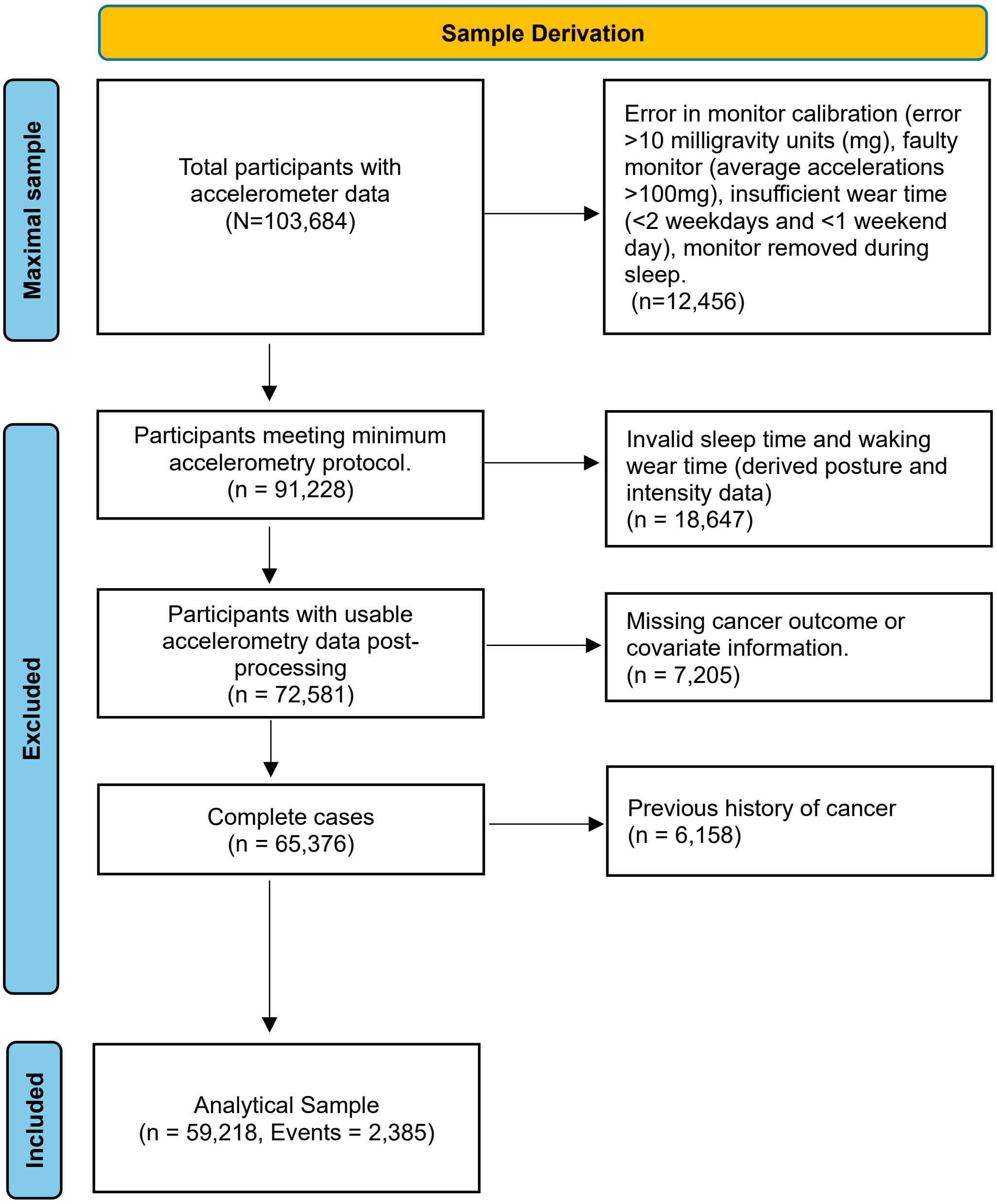

Supplementary Text S1. Classification of intensity and posture from processing algorithms.

Sleep classifier

Sleep duration was assessed with an algorithm, using the absence of change in device tilt angle, developed and validated in 3,752 British participants with sleep diary data and 28 patients with polysomnography data. The sleep period time window derived from the algorithm was 11m and 3m longer compared with a sleep diary in men and women, respectively. The mean c-statistic to detect the sleep period time window compared to polysomnography was 0.86 and 0.83 in clinic-based and healthy sleepers, respectively[1].

Physical activity intensity classifier

Physical activity intensity was classified with a previously adopted two-level Random Forest algorithm[2, 3]. The first level classifies activity type, in contiguous 10s bins, and the second level further sorts the activities into intensity bands. This two-level classifier minimises misclassification between the lower intensity bands as activities are first classified by type, clearly delineating Moving activities, standing utilitarian movements (semi-stationary) and sedentary behaviour (SB) before non-sedentary activities are further classified into intensity bands based on degree of acceleration against gravity. Moving activities are then grouped by their acceleration, as light PA (LPA; <100mg), moderate PA (MPA; ≥100mg) and vigorous (≥400mg). The intensity composition therefore encompassed VPA, MPA, LPA, sedentary time (ST) and sleep.

Posture classifier

Stationary behaviour and physical activity were classified using a previously validated Random Forest (RF) activity classifier. The initial probabilities of activity classification from the RF classifier were temporally smoothed using a Hidden Markov Model (HMM) to generate the final sequence of activity predictions.

Physical activity was categorized in 60-second intervals into five classes: Sitting, Standing Stationary, Standing Moving, Walking/Running, and Cycling and has been shown to have a high degree of classification accuracy[6, 374, 5]. Sleep and non-wear time were distinguished from active periods based on changes in tilt angle and acceleration standard deviation. Accelerometer data were calibrated and orientation-corrected according to established procedures (model specificity and accuracy metrics have been previously outlined by Ahmadi et al., 2024).

Matthew N Ahmadi, Pieter Coenen, Leon Straker, Emmanuel Stamatakis, Device-measured stationary behaviour and cardiovascular and orthostatic circulatory disease incidence, International Journal of Epidemiology, Volume 53, Issue 6, December 2024, dyae136, https://doi.org/10.1093/ije/dyae136

Supplementary Text S2. Isometric log-ratio *'Pivot'* approach to Compositional data analysis.

The pivot approach[6, 7] to compositional data analysis is established for enabling the modelling of highly collinear movement behaviours in tandem. This involves applying the Isometric log-ratio (ILR) transformation to compositional data of *n* components, resulting in *n-1* coordinates which represent all daily movement behaviour in a series of ratios, and which can all be fitted within standard statistical models. This circumvents issues of multicollinearity[6].

The first ILR-coordinate within each model represents the association of one movement behaviour, relative to all others (i.e. vigorous physical activity (VPA) relative to moderate physical activity (MPA), light physical activity (LPA), sedentary time (ST) and sleep duration) with the outcome and is the only interpreted and reported value in each model. The first ILR coefficient represents the expected difference in the outcome when the ILR-coordinate is increased by one unit. The order of the ratios is then re-pivoted, and the model re-fitted, to investigate the association of a different component, relative to all others, with the outcome. Critically, despite interpretability of the direction and consistency of the model coefficient, the magnitude (presented in ILR-transformed units) should be interpreted through isotemporal substitution

**Table S1. ICD Codes / Biobank Var numbers**		
**Variable**	**Coding**	**UKB field**
**Age at baseline**	Continuous (years)	34, 52, accelerometer date-timestamp
**Sex**	Female/Male	31
**Smoker status**	Never, past, current smoker.	20116
**Alcohol Use**	Never drinker, Ex-drinker, within UK guideline levels (<15 weekly units), current above guidelines (≥15 weekly units)).	20117, 1558
**Diet**	Fruits and vegetables servings/day	1309, 1319, 1289, 1299
**Physical-activity related cancer event**	Physical activity-related cancer at 13 sites (oesophageal adenocarcinoma, liver, lung, kidney, gastric cardia, endometrial, myeloid leukaemia, myeloma, colon, head and neck, rectal, bladder, and breast) using ICD-10 classifications: Bladder: C67; Breast: C50; Colon: C18; Endometrial: C54, C55.9; Gastric cardia: C16.0; Head and neck: C00, C11, C12, C13, C14, C30, C31, C32, C33, C38, C39.0, C39.8, C39.9; Kidney: C64.9, C65.9; Liver: C22.0, C22.1; Lung: C34; Myeloid leukaemia: C92; Myeloma: C90.0; Oesophageal adenocarcinoma: C15; Rectal: C19.9, C20.9; Other digestive: C26.0.	100092

**Duration of follow-up (time to event).**	Time from accelerometer assessment to incident cancer diagnosis, death, loss to follow-up, or administrative censoring (in years).	100092
**Prevalent cancer**	Previous cancer at any site (used for exclusion).	20001, 100092
**Prevalent CVD**	Yes/No Identified by self-report and hospitalisation (ICD-10: I0, I11, I13, I20-I51, I60-I69).	20002, 41259
**Education**	College/University; A/AS level;<br>O levels; CSE;<br>NVQ/HND/HNC; other.<br>A/AS = Advanced Placement;<br>O level = High school certificate;<br>CSE = Certificate of secondary education;<br>NVQ/HND/HNC = Vocational qualification / Associates degree.<br>A level, typically at age 18 years;<br>O level, typically at age 16 years;<br>CSE, typically at age 16 years;	6138

**Cholesterol medication use**	Yes/No	6177, 6153
**Blood pressure medication use**	Yes/No	6177, 6153
**Diabetes medication use**	Yes/No	6177, 6153
**Parental history of cancer**	Yes/No Self-reported mother or father diagnosed with cancer	20107, 20110

Table S2. Incident cases of physical-activity (PA)-related cancer during the follow-up period.

PA-related Cancer site	Cases in follow-up period
Breast	823
Colon	355
Lung	301
Bladder	269
Endometrial	119
Head and neck	112
Oesophageal adenocarcinoma	111
Myeloma	94
Liver	65
Myeloid leukaemia	57
Rectal	39
Gastric cardia	24
Kidney	16

Table S3. Competing risk, causes of death.

**Cause of death**	**Deaths (n)**
Cardiovascular disease	451
Other cancer*	400
Respiratory disease	157
Neurological disease	103
Accidents/trauma/external causes	59
Infectious disease	57
Gastrointestinal/liver disease	55
Frailty/nonspecific causes	51
Metabolic/endocrine disease	43
Other	37
Renal/genitourinary disease	34
Rectal Cancer	33
Haematological/immune disease	29
Kidney Cancer	16
Musculoskeletal disease	12
Gastric cardia Cancer	11
Bladder Cancer	*na*[¶]
Endometrial Cancer	*na*[¶]

[¶]Omitted for disclosure control, due to insufficient bin-width. *Other (Non-physical activity-related) cancers (>5% prevalence): Pancreatic (22%), prostate (14%), brain (12%), ovarian (10.2), lymphoma (8.2%).

Figure S2. Raw sample means of (left (A): composition 1 (Posture); right (B): composition 2 (Intensity)

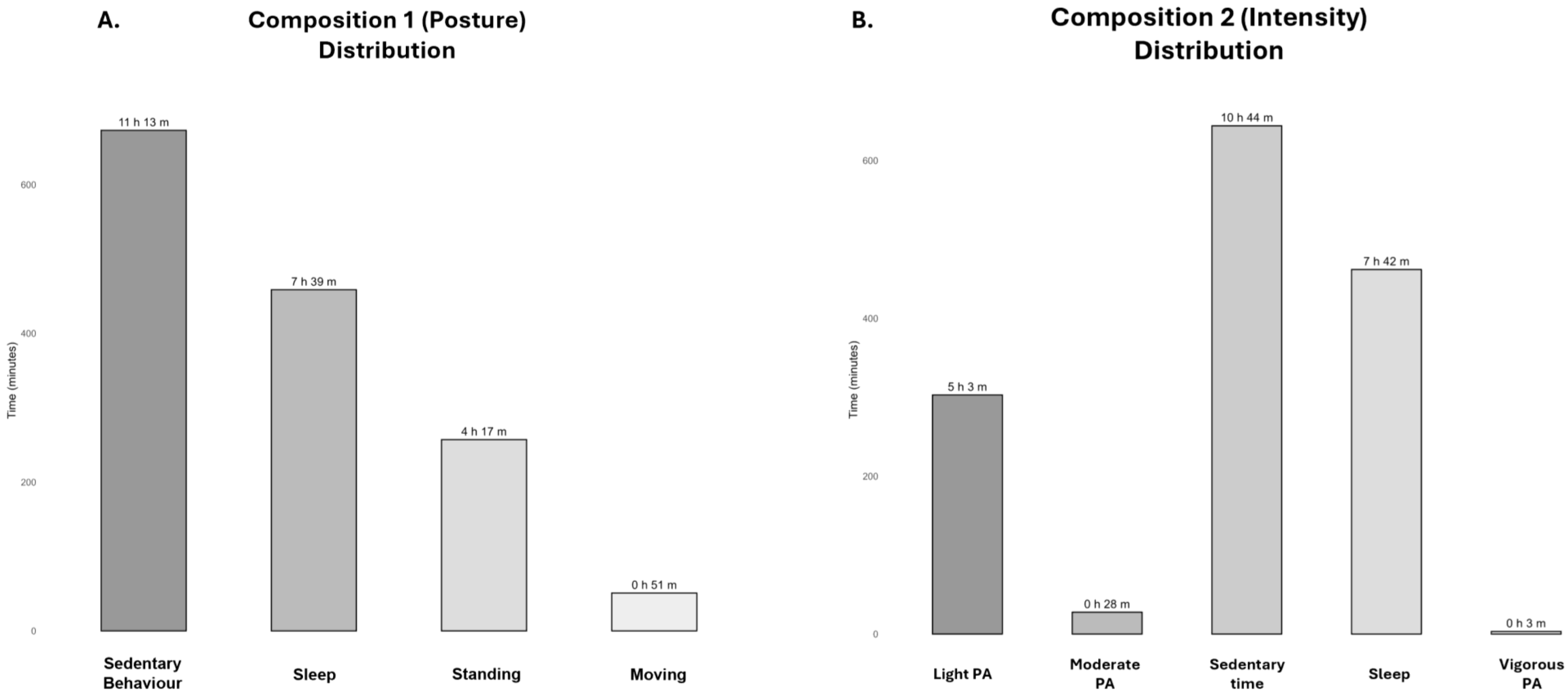

Abbreviations: Hours (h); minutes (m); physical activity (PA).

Table S4. Association of Coordinates of composition 1 (posture) and sleep with risk of physical activity (PA)-related cancers (Fine-Gray Cox Proportional Hazards Model)

	Age and Sex-adjusted				Fully adjusted				Fully adjusted + BMI*			
	HR	95% CI		p-value	HR	95% CI		p-value	HR	95% CI		p-value
**Moving** (vs all other postures)	**0.957**	**0.937**	**0.978**	**<0.001**	**0.962**	**0.942**	**0.983**	**<0.001**	**0.966**	**0.945**	**0.987**	**0.001**
**Standing** (vs all other postures)	**0.911**	**0.872**	**0.953**	**<0.001**	**0.917**	**0.877**	**0.959**	**<0.001**	**0.928**	**0.887**	**1.109**	**0.001**
**Sedentary Behaviour** (vs all other postures)	**1.095**	**1.026**	**1.169**	**0.006**	**1.084**	**1.016**	**1.158**	**0.015**	1.057	0.989	1.130	0.102
Sleep (vs all other postures)	1.047	0.969	1.131	0.241	1.045	0.968	1.129	0.259	1.056	0.977	1.141	0.169

Coefficients are in ILR transformed units and represent the relative association of each behavioural component relative to all other components. While direction of association can be interpreted, magnitude should be interpreted from isotemporal substitution analysis.. Bold: Significant at p<0.05. HR: Hazard Ratio. CI: Confidence interval BMI: Body mass index. *n=59,129, 2383 events.

Table S5 Predicted Hazard Ratio (HR) from theoretical isotemporal substitution around sample average of Moving whilst holding other components constant at sample average.

Reallocation of time between behaviours	**+3 min**			**+5 min**			**+10 min**			**+15 min**			**+30 min**			**+45 min**			**+60 min**			**+90 min**		
	**HR**	**95% CI**		**HR**	**95% CI**		**HR**	**95% CI**		**HR**	**95% CI**		**HR**	**95% CI**		**HR**	**95% CI**		**HR**	**95% CI**		**HR**	**95% CI**	
Reallocating **Sedentary behaviour to Moving** (centred at the average day: 51m moving, 11h 13min sedentary behaviour)	**1.00**	0.99	1.00	**0.99**	0.99	1.00	**0.98**	0.98	0.99	**0.98**	0.97	0.99	**0.96**	0.94	0.98	**0.94**	0.92	0.97	**0.93**	0.90	0.96	**0.91**	0.87	0.95
Reallocating **Standing to Moving** (centred at the average day: 51m moving, 4h 17 min standing)	1.00	1.00	1.00	1.00	0.99	1.00	0.99	0.98	1.00	0.99	0.98	1.00	0.99	0.96	1.01	0.98	0.95	1.02	0.99	0.95	1.03	1.00	0.94	1.06
Reallocating **Sleep to Moving** (centred at the average day: 51m moving, 7h 39 min sleep)	**1.00**	0.99	1.00	**0.99**	0.99	1.00	**0.98**	0.98	0.99	**0.98**	0.97	0.99	**0.96**	0.94	0.98	**0.95**	0.91	0.98	**0.93**	0.89	0.97	**0.91**	0.86	0.96
Reallocating **Moving to Sedentary behaviour** (centred at the average day: 51m moving, 11h 13min sedentary behaviour)	**1.01**	1.00	1.01	**1.01**	1.00	1.01	**1.02**	1.01	1.03	**1.03**	1.01	1.03	**1.08**	1.04	1.12	**1.19**	1.08	1.30	-	-	-	-	-	-
Reallocating **Moving to Standing** (centred at the average day: 51m moving, 4h 17 min standing)	**1.00**	1.00	1.01	**1.00**	1.00	1.01	**1.01**	1.00	1.02	**1.02**	1.00	1.02	**1.05**	1.01	1.09	**1.14**	1.04	1.25	-	-	-	-	-	-
Reallocating **Moving to Sleep** (centred at the average day: 51m moving, 7h 39 min sleep)	**1.01**	1.00	1.01	**1.01**	1.00	1.01	**1.02**	1.01	1.03	**1.03**	1.01	1.03	**1.07**	1.03	1.12	**1.18**	1.08	1.30	-	-	-	-	-	-

Abbreviations: HR: Hazard Ratio; CI: Confidence interval; min: minutes. h: Hours. Omitted estimates are outside of limits of prediction/theoretical reallocation.

Table S6 Predicted Hazard Ratio (HR) from Isotemporal substitution around sample average of standing whilst holding other components constant at sample average.

Reallocation of time between behaviours	**+3 min**			**+5 min**			**+10 min**			**+15 min**			**+30 min**			**+45 min**			**+60 min**			**+90 min**		
	**HR**	**95% CI**		**HR**	**95% CI**		**HR**	**95% CI**		**HR**	**95% CI**		**HR**	**95% CI**		**HR**	**95% CI**		**HR**	**95% CI**		**HR**	**95% CI**	
Reallocating **Sedentary behaviour to Standing** (centred at the average day: 4h 17 min standing, 11h 13min sedentary behaviour)	**1.00**	1.00	1.00	**1.00**	0.99	1.00	**0.99**	0.99	1.00	**0.99**	0.98	0.99	**0.97**	0.96	0.99	**0.96**	0.95	0.98	**0.95**	0.93	0.97	**0.93**	0.90	0.96
Reallocating **Moving to Standing** (centred at the average day: 51m moving, 4h 17 min standing)	1.00	1.00	1.01	1.00	1.00	1.01	1.01	1.00	1.02	**1.02**	1.00	1.03	**1.05**	1.01	1.09	**1.14**	1.04	1.25	-	-	-	-	-	-
Reallocating **Sleep to Standing** (centred at the average day: 4h 17 min standing, 7h 39 min sleep)	**1.00**	1.00	1.00	**1.00**	0.99	1.00	**0.99**	0.99	1.00	**0.99**	0.98	1.00	**0.98**	0.96	0.99	**0.96**	0.94	0.99	**0.95**	0.92	0.99	**0.93**	0.89	0.98
Reallocating **Standing to Sedentary behaviour** (centred at the average day: 4h 17 min standing, 11h 13min sedentary behaviour)	1.00	1.00	1.00	1.00	1.00	1.01	**1.01**	1.00	1.01	**1.01**	1.01	1.02	**1.03**	1.02	1.04	**1.04**	1.02	1.06	**1.06**	1.03	1.09	**1.10**	1.05	1.14
Reallocating **Standing to Moving** (centred at the average day: 51m moving, 4h 17 min standing)	**1.00**	1.00	1.00	**1.00**	0.99	1.00	**0.99**	0.98	1.00	**0.99**	0.98	1.00	0.99	0.96	1.01	0.98	0.95	1.02	0.99	0.95	1.03	1.00	0.94	1.06
Reallocating **Standing to Sleep** (centred at the average day: 4h 17 min standing, 7h 39 min sleep)	**1.00**	1.00	1.00	**1.00**	1.00	1.01	**1.01**	1.00	1.01	**1.01**	1.00	1.02	**1.03**	1.01	1.04	**1.04**	1.01	1.07	**1.06**	1.02	1.09	**1.09**	1.03	1.15

Abbreviations: HR: Hazard Ratio; CI: Confidence interval; min: minutes. h: Hours. Omitted estimates are outside of limits of prediction/theoretical reallocation. **Bold**: Significant at $p<0.05$

Table S7 Predicted Hazard Ratio (HR) from Isotemporal substitution around sample average of Sedentary Behaviour postures whilst holding other components constant at sample average.

Reallocation of time between behaviours	**+3 min**			**+5 min**			**+10 min**			**+15 min**			**+30 min**			**+45 min**			**+60 min**			**+90 min**		
	**HR**	**95% CI**		**HR**	**95% CI**		**HR**	**95% CI**		**HR**	**95% CI**		**HR**	**95% CI**		**HR**	**95% CI**		**HR**	**95% CI**		**HR**	**95% CI**	
Reallocating **Standing to Sedentary behaviour** (centred at the average day: 4h 17 min standing, 11h 13min sedentary behaviour)	1.00	1.00	1.00	1.00	1.00	1.01	**1.01**	1.00	1.01	**1.03**	1.02	1.04	**1.04**	1.02	1.06	**1.06**	1.03	1.09	**-**	-	-	**-**	-	-
Reallocating **Moving to Sedentary behaviour** (centred at the average day: 51m moving, 11h 13min sedentary behaviour)	1.01	1.00	1.01	1.01	1.00	1.01	**1.02**	1.01	1.03	**1.03**	1.01	1.03	**1.08**	1.04	1.12	**1.19**	1.08	1.30	-	-	-	-	-	-
Reallocating **Sleep to Sedentary behaviour** (centred at the average day: 11h 13min sedentary behaviour, 7h 39 min sleep)	1.00	1.00	1.00	1.00	1.00	1.00	1.00	1.00	1.01	1.00	0.99	1.01	1.00	0.99	1.02	1.00	0.98	1.02	1.00	0.97	1.03	1.00	0.96	1.05
Reallocating **Sedentary behaviour to Standing** (centred at the average day: 4h 17 min standing, 11h 13min sedentary behaviour)	**1.00**	1.00	1.00	**1.00**	0.99	1.00	**0.99**	0.99	1.00	**0.99**	0.98	0.99	**0.97**	0.96	0.99	**0.96**	0.95	0.98	**0.95**	0.93	0.97	**0.93**	0.90	0.96
Reallocating **Sedentary behaviour to Moving** (centred at the average day: 51m moving, 11h 13min sedentary behaviour)	**1.00**	0.99	1.00	**0.99**	0.99	1.00	**0.98**	0.98	0.99	**0.98**	0.97	0.99	**0.96**	0.94	0.98	**0.94**	0.92	0.97	**0.93**	0.90	0.96	**0.91**	0.87	0.95

Reallocating **Standing to Sleep** (centred at the average day: 11h 13min sedentary behaviour, 7h 39 min sleep)	1.00	1.00	1.00	1.00	1.00	1.00	1.00	1.00	0.99	1.00	0.99	1.01	1.00	0.98	1.01	1.00	0.98	1.02	1.00	0.97	1.02	0.99	0.95	1.04

Abbreviations: HR: Hazard Ratio; CI: Confidence interval; min: minutes. h: Hours. Omitted estimates are outside of limits of prediction/theoretical reallocation. **Bold:** Significant at p<0.05

Table S8. Association of Coordinates of composition 2 (intensity) and sleep with risk of physical activity (PA)-related cancers (Fine-Gray Cox Proportional Hazards Model)

	Age and Sex-adjusted				Fully adjusted				Fully adjusted + BMI			
	HR	95% CI		p-value	HR	95% CI		p-value	HR	95% CI		p-value
**Vigorous PA** (vs all other behaviours)	**0.922**	**0.878**	**0.968**	**0.001**	**0.932**	**0.887**	**0.978**	**0.004**	**0.945**	**0.899**	**0.993**	**0.026**
Moderate PA (vs all other behaviours)	0.971	0.903	1.043	0.415	0.973	0.905	1.046	0.454	0.978	0.91	1.051	0.546
**Light PA** (vs all other behaviours)	**0.781**	**0.642**	**0.95**	**0.013**	**0.792**	**0.651**	**0.965**	**0.02**	**0.802**	**0.659**	**0.976**	**0.027**
Sleep (vs all other behaviours)	1.169	0.948	1.442	0.145	1.165	0.945	1.437	0.152	1.201	0.973	1.483	0.088
Sedentary time (vs all other behaviours)	1.225	0.993	1.51	0.058	1.195	0.969	1.475	0.096	1.123	0.907	1.39	0.286

Coefficients are in ILR transformed units and represent the relative association of each behavioural component relative to all other components. While direction of association can be interpreted, magnitude should be interpreted from isotemporal substitution analysis.. Bold: Significant at p<0.05. HR: Hazard Ratio; CI: Confidence interval. BMI: Body mass index.. *n=59,129, 2383 events.

Table S9. Predicted HR from Isotemporal substitution around sample average of VPA whilst holding other components constant at sample average.

Reallocation of time between behaviours	**+3 min**			**+5 min**			**+10 min**			**+15 min**			**+30 min**			**+45 min**			**+60 min**			**+90 min**		
	**HR**	**95% CI**		**HR**	**95% CI**		**HR**	**95% CI**		**HR**	**95% CI**		**HR**	**95% CI**		**HR**	**95% CI**		**HR**	**95% CI**		**HR**	**95% CI**	
Reallocating **Sedentary time to Vigorous PA** (centred at the average day: 3 min VPA, 10h 44 min sedentary time)	**0.96**	0.93	0.99	**0.94**	0.90	0.98	**0.91**	0.86	0.97	**0.89**	0.83	0.96	**0.86**	0.77	0.95	**0.83**	0.74	0.94	**0.82**	0.72	0.93	**0.79**	0.68	0.91
Reallocating **Moderate PA to Vigorous PA** (centred at the average day: 3 min VPA, 28 min MPA)	**0.96**	0.93	0.99	**0.95**	0.90	0.99	0.92	0.85	1.00	0.91	0.82	1.02	-	-	-	-	-	-	-	-	-	-	-	-
Reallocating **Light PA to Vigorous PA** (centred at the average day: 3 min VPA, 5h 3min LPA)	**0.96**	0.93	0.99	**0.95**	0.91	0.99	**0.92**	0.87	0.98	**0.91**	0.84	0.98	**0.88**	0.79	0.98	**0.87**	0.77	0.99	0.87	0.76	1.00	0.87	0.74	1.02
Reallocating **Sleep to Vigorous PA** (centred at the average day: 3 min VPA, 7h 42 min sleep)	**0.96**	0.93	0.99	**0.94**	0.90	0.98	**0.91**	0.86	0.97	**0.89**	0.83	0.96	**0.85**	0.77	0.95	**0.83**	0.74	0.94	**0.81**	0.71	0.93	**0.78**	0.67	0.91
Reallocating **Vigorous PA to Sedentary time** (centred at the average day: 3 min VPA, 10h 44 min sedentary time)	**1.17**	1.05	1.29																					
Reallocating **Vigorous PA to Moderate PA** (centred at the average day: 3 min VPA, 28 min MPA)	**1.16**	1.05	1.29	-	-	-	-	-	-	-	-	-	-	-	-	-	-	-	-	-	-	-	-	-
Reallocating **Vigorous PA to Light PA** (centred at the average day: 3 min VPA, 5h 3min LPA)	**1.16**	1.05	1.29	-	-	-	-	-	-	-	-	-	-	-	-	-	-	-	-	-	-	-	-	-

Reallocating **Vigorous PA to Sleep** (centred at the average day: 3 min VPA, 7h 42 min sleep)	**1.17**	1.05	1.29	-	-	-	-	-	-	-	-	-	-	-	-	-	-	-	-	-	-	-	-	-

Abbreviations: PA: Physical Activity; HR: Hazard Ratio; CI: Confidence interval; min: minutes. h: Hours. Omitted estimates are outside of limits of prediction/theoretical reallocation. **Bold:** Significant at p<0.05

Table S10. Predicted HR from Isotemporal substitution around sample average of LPA whilst holding other components constant at sample average.

Reallocation of time between behaviours	**+3 min**			**+5 min**			**+10 min**			**+15 min**			**+30 min**			**+45 min**			**+60 min**			**+90 min**		
	**HR**	**95% CI**		**HR**	**95% CI**		**HR**	**95% CI**		**HR**	**95% CI**		**HR**	**95% CI**		**HR**	**95% CI**		**HR**	**95% CI**		**HR**	**95% CI**	
Reallocating **Sedentary time to Light PA** (centred at the average day: 5h 3min LPA, 10h 44 min sedentary time)	**1.00**	0.99	1.00	**1.00**	0.99	1.00	**0.99**	0.98	1.00	**0.99**	0.98	1.00	**0.97**	0.95	0.99	**0.96**	0.93	0.99	**0.95**	0.91	0.99	**0.92**	0.87	0.99
Reallocating **Moderate PA to Light PA** (centred at the average day: 5h 3min LPA, 28 min MPA)	1.00	0.99	1.02	1.00	0.97	1.04	1.01	0.96	1.06	-	-	-	-	-	-	-	-	-	-	-	-	-	-	-
Reallocating **Vigorous PA to Light PA** (centred at the average day: 3 min VPA, 5h 3min LPA)	**1.16**	1.05	1.29	-	-	-	-	-	-	-	-	-	-	-	-	-	-	-	-	-	-	-	-	-
Reallocating **Sleep to Light PA** (centred at the average day: 5h 3min LPA, 7h 42 min sleep)	**1.00**	0.99	1.00	**1.00**	0.99	1.00	**0.99**	0.98	1.00	**0.99**	0.97	1.00	**0.97**	0.95	1.00	**0.96**	0.92	0.99	**0.94**	0.90	0.99	**0.92**	0.86	0.99
Reallocating **Light PA to Sedentary time** (centred at the average day: 5h 3min LPA, 10h 44 min sedentary time)	**1.00**	1.00	1.01	**1.00**	1.00	1.01	**1.01**	1.00	1.02	**1.01**	1.00	1.03	**1.03**	1.01	1.05	**1.05**	1.01	1.08	**1.06**	1.01	1.12	**1.10**	1.02	1.19
Reallocating **Light PA to Moderate PA** (centred at the average day: 5h 3min LPA, 28 min MPA)	1.00	0.99	1.01	1.00	0.99	1.01	1.00	0.98	1.02	1.00	0.97	1.03	1.00	0.95	1.06	1.01	0.94	1.09	1.02	0.93	1.12	1.04	0.91	1.18
Reallocating **Light PA to Vigorous PA** (centred at the average day: 3 min VPA, 5h 3min LPA)	**0.96**	0.93	0.99	**0.96**	0.93	0.99	**0.95**	0.91	0.99	**0.92**	0.87	0.98	**0.88**	0.79	0.98	**0.87**	0.77	0.99	**0.87**	0.76	1.00	0.87	0.74	1.02

Reallocating **Light PA to Sleep** (centred at the average day: 5h 3min LPA, 7h 42 min sleep)	**1.00**	1.00	1.01	**1.00**	1.00	1.01	**1.01**	1.00	1.02	**1.02**	1.00	1.03	**1.03**	1.01	1.06	**1.05**	1.01	1.09	**1.06**	1.01	1.12	**1.10**	1.02	1.19

Abbreviations: PA: Physical Activity; HR: Hazard Ratio; CI: Confidence interval; min: minutes. h: Hours. Omitted estimates are outside of limits of prediction/theoretical reallocation. **Bold:** Significant at $p<0.05$

**Table S11 Association of Coordinates of daily-movement behaviours (intensities, including MVPA) and sleep with risk of PA-related cancers (Fine-Gray Cox Proportional Hazards Model) (including all events).**								
	**Age and Sex-adjusted**				**Fully adjusted**			
	**HR**	**95% CI**		**p-value**	**HR**	**95% CI**		**p-value**
**Moderate-to-Vigorous PA** (vs all other behaviours)	**0.950**	**0.923**	**0.979**	**0.001**	**0.956**	**0.928**	**0.985**	**0.003**
**Light PA** (vs all other behaviours)	**0.891**	**0.813**	**0.975**	**0.012**	**0.897**	**0.819**	**0.983**	**0.019**
Sleep (vs all other behaviours)	1.073	0.974	1.182	0.155	1.072	0.973	1.181	0.162
Sedentary time (vs all other behaviours)	1.101	0.999	1.213	0.052	1.088	0.987	1.199	0.090

Coefficients are in ILR transformed units and represent the relative association of each behavioural component relative to all other components. While direction of association can be interpreted, magnitude should be interpreted from isotemporal substitution analysis.. Bold: Significant at p<0.05. HR: Hazard Ratio. CI: Confidence interval.

Figure S3. Association of ratio variables (proportion of vigorous physical activity (VPA) and moderate physical activity (MPA) within moderate-to-vigorous physical activity (MVPA)) with risk of PA-related cancers (Age, sex, MVPA and sedentary-behaviour-adjusted Fine-Gray Cox Proportional Hazards Model, with knots placed at the 10th, 50th and, 90th percentile).

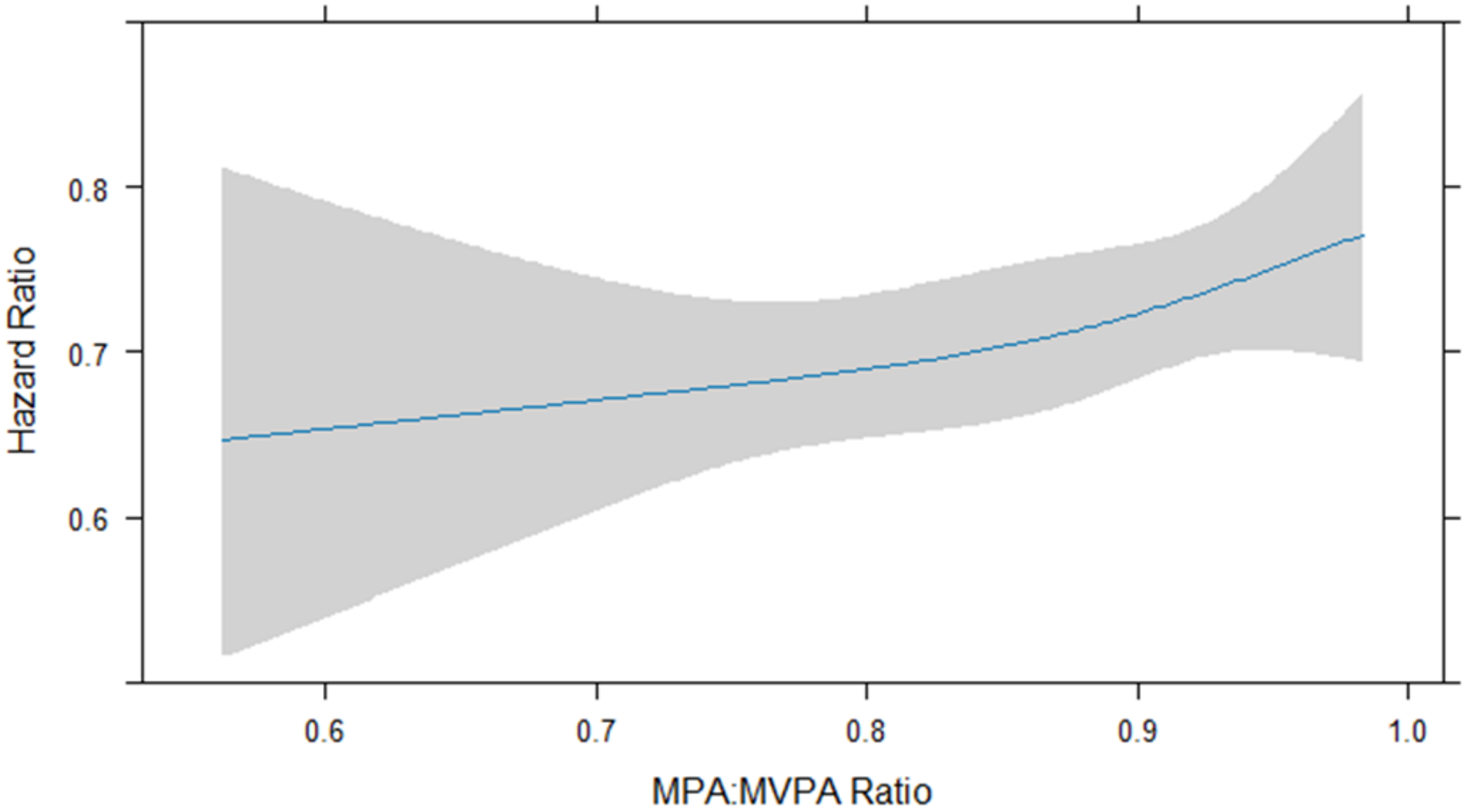

Adjusted to: MVPA= 34m; SB=10h 36m; Sex=Male; Age=62y

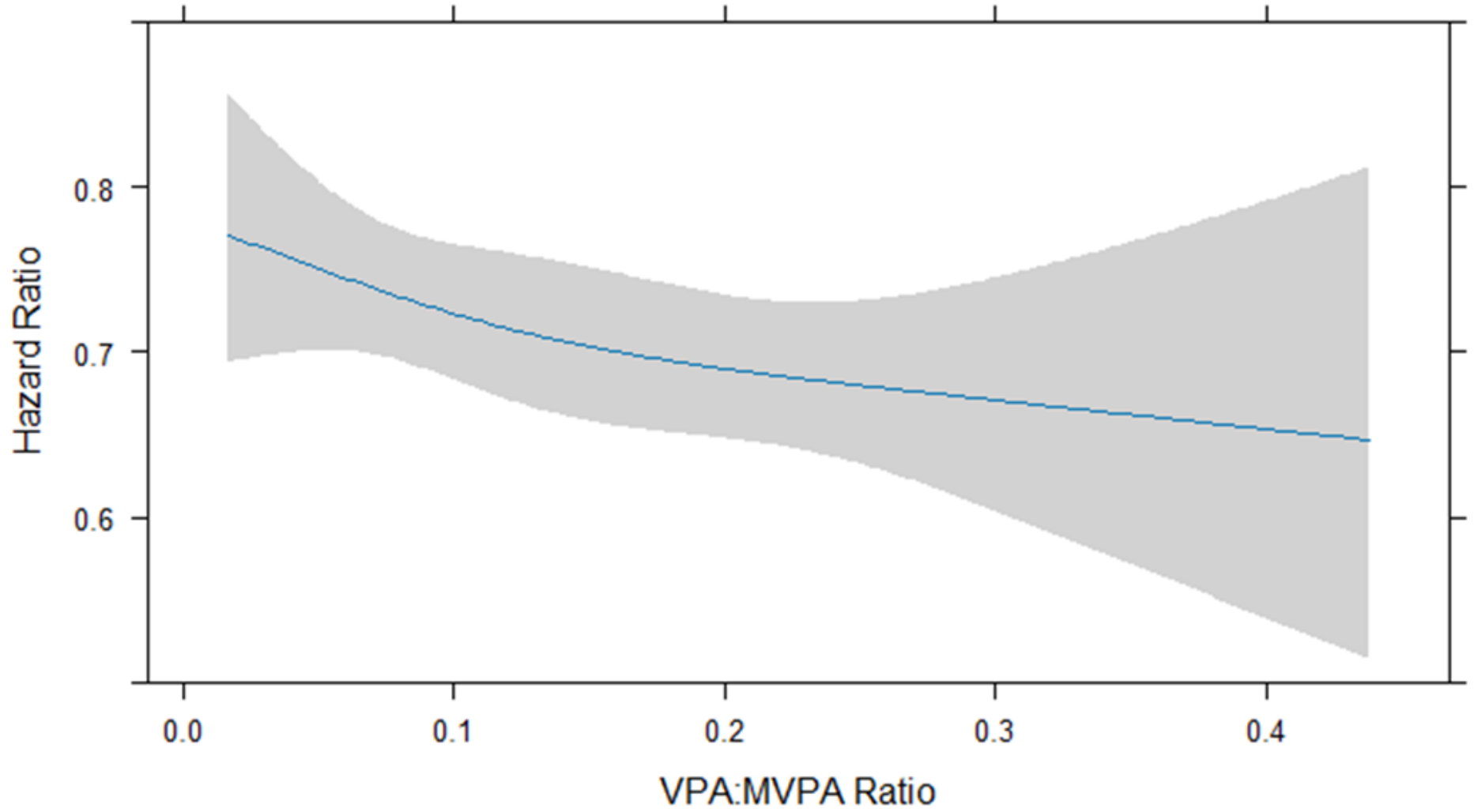

Adjusted to: MVPA= 34m; SB=10h 36m; Sex=Male; Age=62y

**Table S12. E-values of posture model**		
Exposure	E-value	(Lower-limit)
Moving (vs other postures)	1.242	(1.151)
Standing (vs other postures)	1.318	(1.204)
Sedentary Behaviour (vs other postures)	1.305	(1.116)
Sleep (vs other postures)	Null	-

Point estimate (and upper limit of the confidence interval in brackets) that an unmeasured confounder must have with both the exposure and outcome (conditional on the measured covariates) to render the exposure-outcome association null.

**Table S13. E-values of Intensity model**		
Exposure	E-value	(Lower-limit)
Vigorous Physical Activity (vs other movement behaviours)	1.282	(1.143)
Moderate Physical Activity (vs other movement behaviours)	Null	-
Light Physical Activity (vs other movement behaviours)	1.628	(1.187)
Sedentary time (vs other movement behaviours)	Null	-
Sleep (vs other movement behaviours)	Null	-

Point estimate (and upper limit of the confidence interval in brackets) that an unmeasured confounder must have with both the exposure and outcome (conditional on the measured covariates) to render the exposure-outcome association null.

Table S14. Association of Coordinates of daily-movement behaviours (postures) and sleep with risk of physical activity (PA)-related cancers (Fine-Gray Cox Proportional Hazards Model) with removal of first 1 year of events.

	Age and Sex-adjusted				Fully adjusted			
	**HR**	**95% CI**		**p-value**	**HR**	**95% CI**		**p-value**
**Moving** (vs all other postures)	**0.960**	**0.939**	**0.981**	**<0.001**	**1.034**	**0.941**	**0.987**	**0.002**
**Standing** (vs all other postures)	**0.917**	**0.875**	**0.961**	**<0.001**	**0.923**	**0.881**	**0.968**	**<0.001**
**Sedentary Behaviour** (vs all other postures)	**1.088**	**1.015**	**1.164**	**0.017**	**1.075**	**1.003**	**1.151**	**0.040**
Sleep (vs all other postures)	1.045	0.961	1.134	0.285	1.044	0.963	1.132	0.300

Coefficients are in ILR transformed units and represent the relative association of each behavioural component relative to all other components. While direction of association can be interpreted, magnitude should be interpreted from isotemporal substitution analysis.. Bold: Significant at p<0.05. HR: Hazard Ratio.

Table S15. Association of Coordinates of daily-movement behaviours (intensities) and sleep with risk of physical activity (PA)-related cancers (Fine-Gray Cox Proportional Hazards Model) with removal of first 1 year of events.

	Age and Sex-adjusted				Fully adjusted			
	HR	95% CI		p-value	HR	95% CI		p-value
**Vigorous PA** (vs all other behaviours)	**0.936**	**0.890**	**0.985**	**0.018**	**0.946**	**0.899**	**0.997**	**0.037**
Moderate PA (vs all other behaviours)	0.959	0.889	1.035	0.277	0.962	0.891	1.038	0.319
Light PA (vs all other behaviours)	1.156	0.927	1.443	0.199	1.125	0.901	1.405	0.299
**Sleep** (vs all other behaviours)	**0.795**	**0.795**	**0.647**	**0.030**	**0.807**	**0.807**	**0.656**	**0.042**
Sedentary time (vs all other behaviours)	1.212	0.969	1.513	0.091	1.210	0.969	1.511	0.093

Coefficients are in ILR transformed units and represent the relative association of each behavioural component relative to all other components. While direction of association can be interpreted, magnitude should be interpreted from isotemporal substitution analysis.. Bold: Significant at p<0.05. HR: Hazard Ratio.

Table S16. Association of Coordinates of daily-movement behaviours (postures) and sleep with risk of physical activity (PA)-related cancers (Fine-Gray Cox Proportional Hazards Model) with removal of first 2 years events).

	**Age and Sex-adjusted**				**Fully adjusted**			
	**HR**	**95% CI**		**p-value**	**HR**	**95% CI**		**p-value**
**Moving** (vs all other postures)	**0.954**	**0.932**	**0.978**	**0.000**	**0.960**	**0.937**	**0.984**	**0.001**
**Standing** (vs all other postures)	**0.920**	**0.875**	**0.967**	**0.001**	**0.926**	**0.880**	**0.974**	**0.003**
**Sedentary Behaviour** (vs all other postures)	**1.088**	**1.010**	**1.172**	**0.026**	**1.084**	**1.016**	**1.158**	**0.015**
Sleep (vs all other postures)	1.047	0.969	1.042	0.134	1.044	0.957	1.140	0.331

Coefficients are in ILR transformed units and represent the relative association of each behavioural component relative to all other components. While direction of association can be interpreted, magnitude should be interpreted from isotemporal substitution analysis.. Bold: Significant at p<0.05. HR: Hazard Ratio.

Table S17. Association of Coordinates of daily-movement behaviours (intensities) and sleep with risk of physical activity (PA)-related cancers (Fine-Gray Cox Proportional Hazards Model) (after removal of first 2 years of events).

	Age and Sex-adjusted				Fully adjusted			
	**HR**	**95% CI**		**p-value**	**HR**	**95% CI**		**p-value**
Vigorous Physical activity (vs all other behaviours)	0.948	0.897	1.001	0.056	0.958	0.906	1.012	0.126
Moderate Physical activity (vs all other behaviours)	0.937	0.863	1.016	0.116	0.940	0.866	1.020	0.135
**Light Physical activity** (vs all other behaviours)	**0.759**	**0.607**	**0.949**	**0.015**	**0.771**	**0.616**	**0.963**	**0.022**
Sleep (vs all other behaviours)	1.226	0.965	1.557	0.096	1.220	0.961	1.550	0.102
Sedentary time (vs all other behaviours)	1.211	0.954	1.537	0.116	1.182	0.930	1.501	0.172

Coefficients are in ILR transformed units and represent the relative association of each behavioural component relative to all other components. While direction of association can be interpreted, magnitude should be interpreted from isotemporal substitution analysis.. Bold: Significant at p<0.05. HR: Hazard Ratio.

Figure S4. Difference in composition 2 (intensity) of individuals with physical activity-related cancer incidence within first 1 year follow-up, relative to sample average.

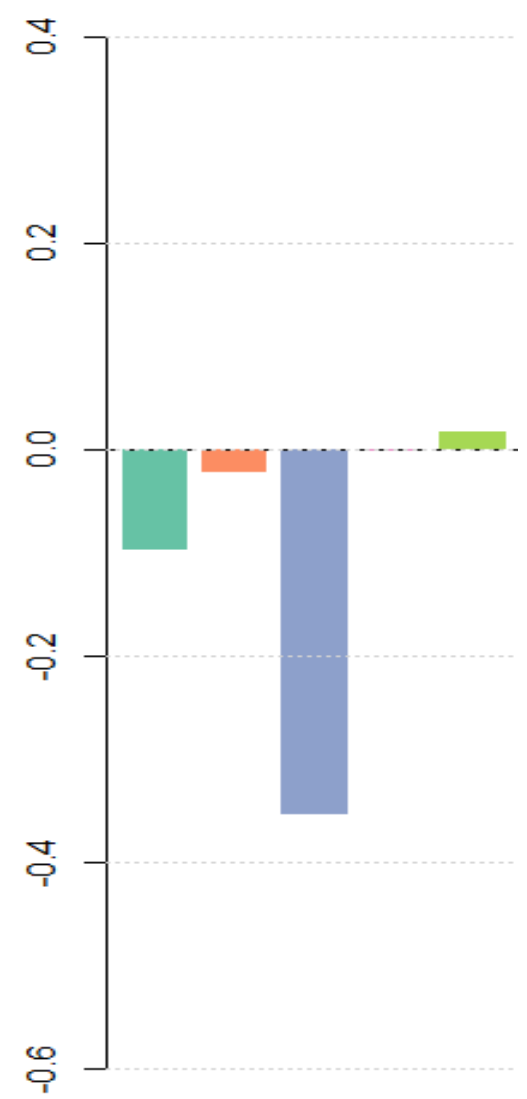

Vigorous Physical activity	Sleep duration	Moderate Physical activity	Light Physical activity	Sedentary time
0h 2 min	7h 40 min	23 min	4h 54 min	11h 0 min

Figure S5. Difference in composition 2 (intensity) of individuals with physical activity-related cancer incidence excluding first 2 year follow-up, relative to sample average.

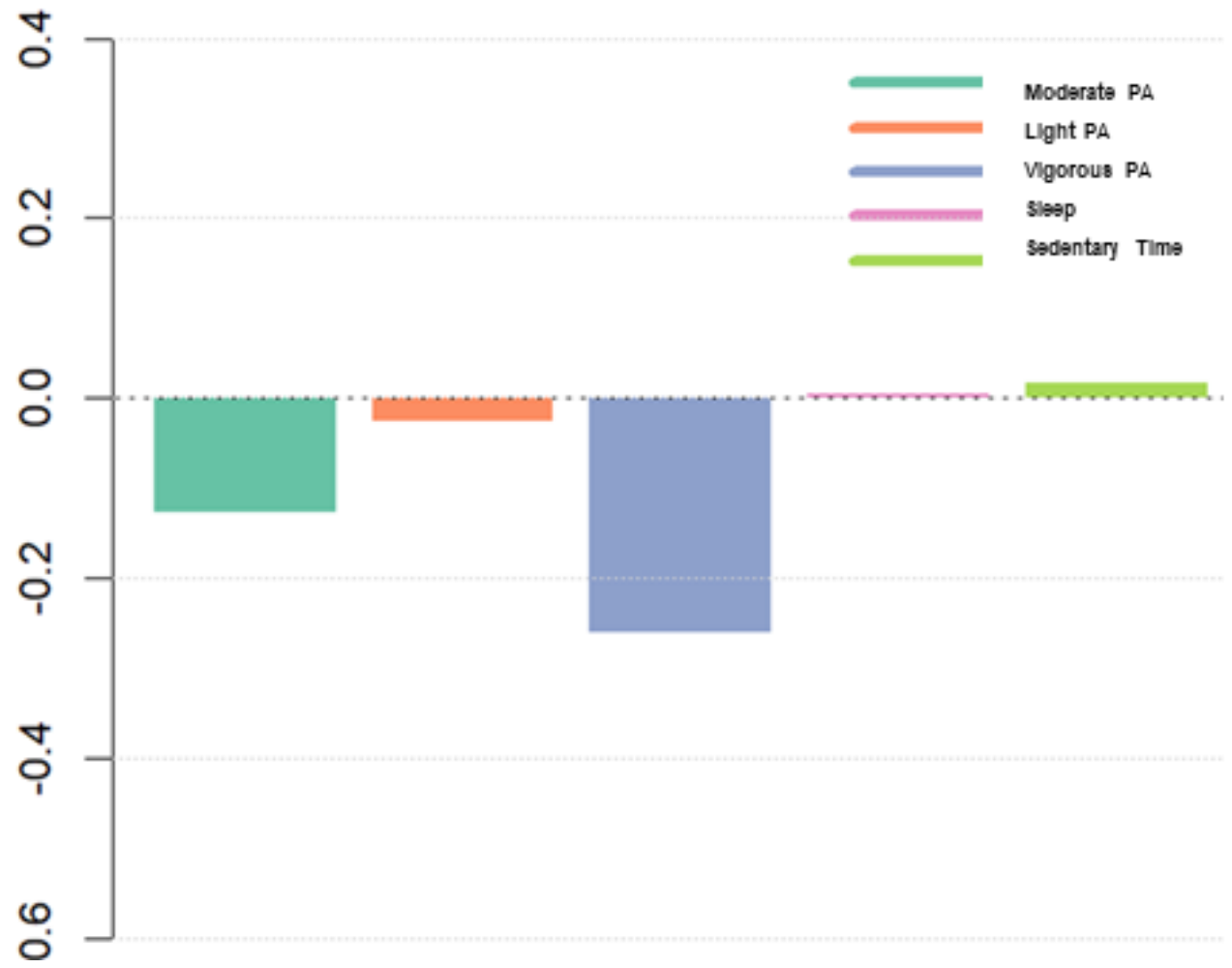

Vigorous Physical activity*	Sleep duration	Moderate Physical activity	Light Physical activity	Sedentary time
0h 3 min	7h 44 min	25 min	4h 55 min	10h 54 min

MANOVA Pillai's trace test of multivariate differences in compositional centre between PA-related cancer in first 2y follow-up compared to outside of 2y follow-up (p=0.2405)

*Significant t-test on difference in compositional average VPA between PA-related cancer in first 2y follow-up compared to outside of 2y follow-up (p<0.0177).

**Table S18. Association of Coordinates of daily-movement behaviours (intensities, including moderate-to-vigorous physical activity (MVPA) and sleep with risk of physical activity (PA)-related cancers (Fine-Gray Cox Proportional Hazards Model) (after removal of first 2 years of events).**								
	**Age and Sex-adjusted**				**Fully adjusted**			
	**HR**	**95% CI**		**p-value**	**HR**	**95% CI**		**p-value**
**Moderate-to-Vigorous PA** (vs all other behaviours)	**0.946**	**0.914**	**0.977**	**0.001**	**0.952**	**0.92**	**0.985**	**0.0038**
**Light PA** (vs all other behaviours)	**0.881**	**0.772**	**1.006**	**0.0159**	**0.888**	**0.805**	**0.979**	**0.0232**
Sleep (vs all other behaviours)	1.09	0.98	1.22	0.1139	1.08	0.97	1.21	0.1719
Sedentary time (vs all other behaviours)	1.098	0.973	1.241	0.0967	1.096	0.983	1.211	0.1028

Coefficients are in ILR transformed units and represent the relative association of each behavioural component relative to all other components. While direction of association can be interpreted, magnitude should be interpreted from isotemporal substitution analysis.. Bold: Significant at p<0.05. HR: Hazard Ratio.

Figure S6. Associations of each composition 1 (posture) component (relative to all other movement behaviour) with physical activity (PA)-relatedcancer, stratified by site.

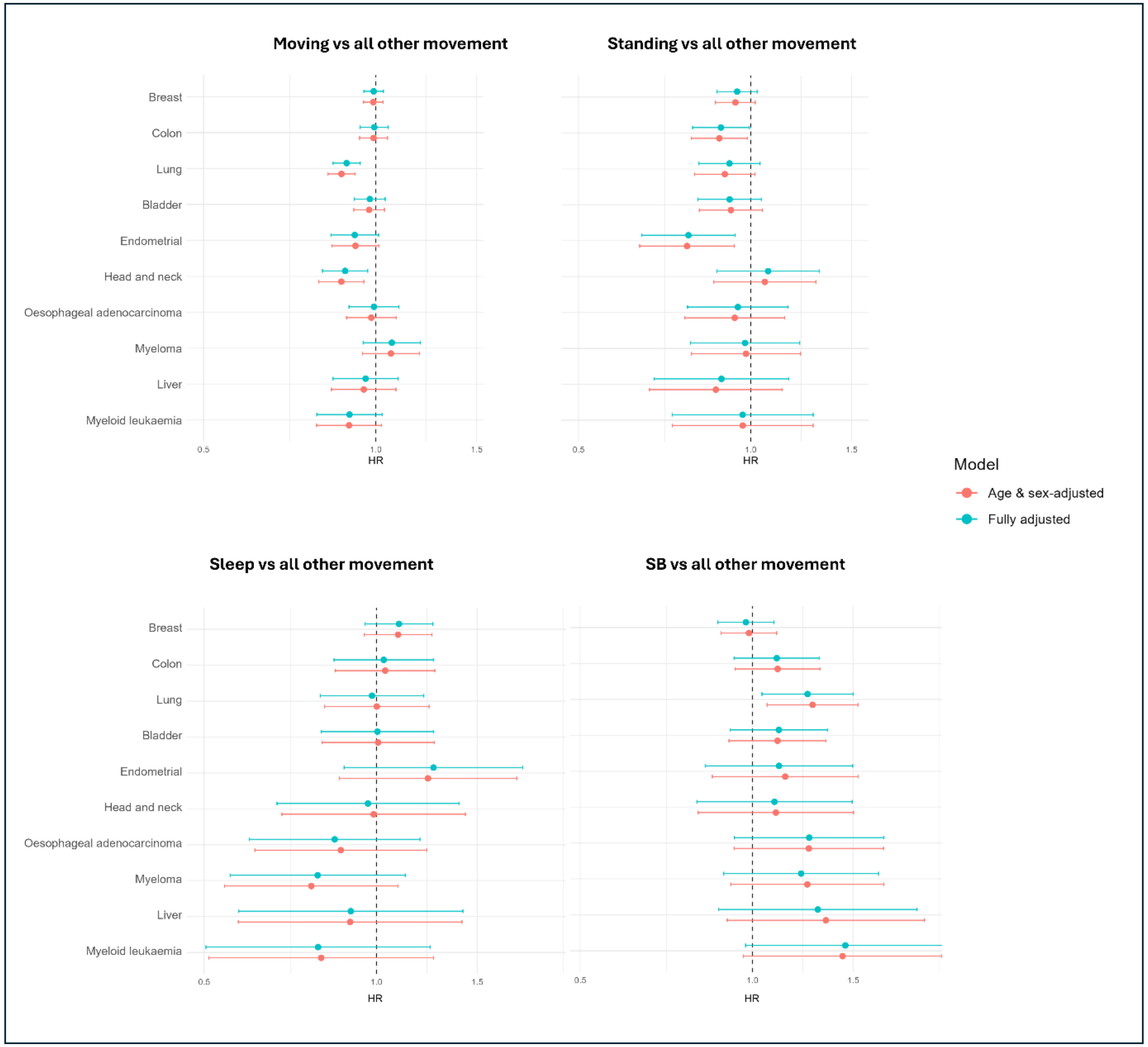

Figure S7. Associations of each composition 2 (intensity) component (relative to all other movement behaviour) with physical activity (PA)-related cancer, stratified by site.

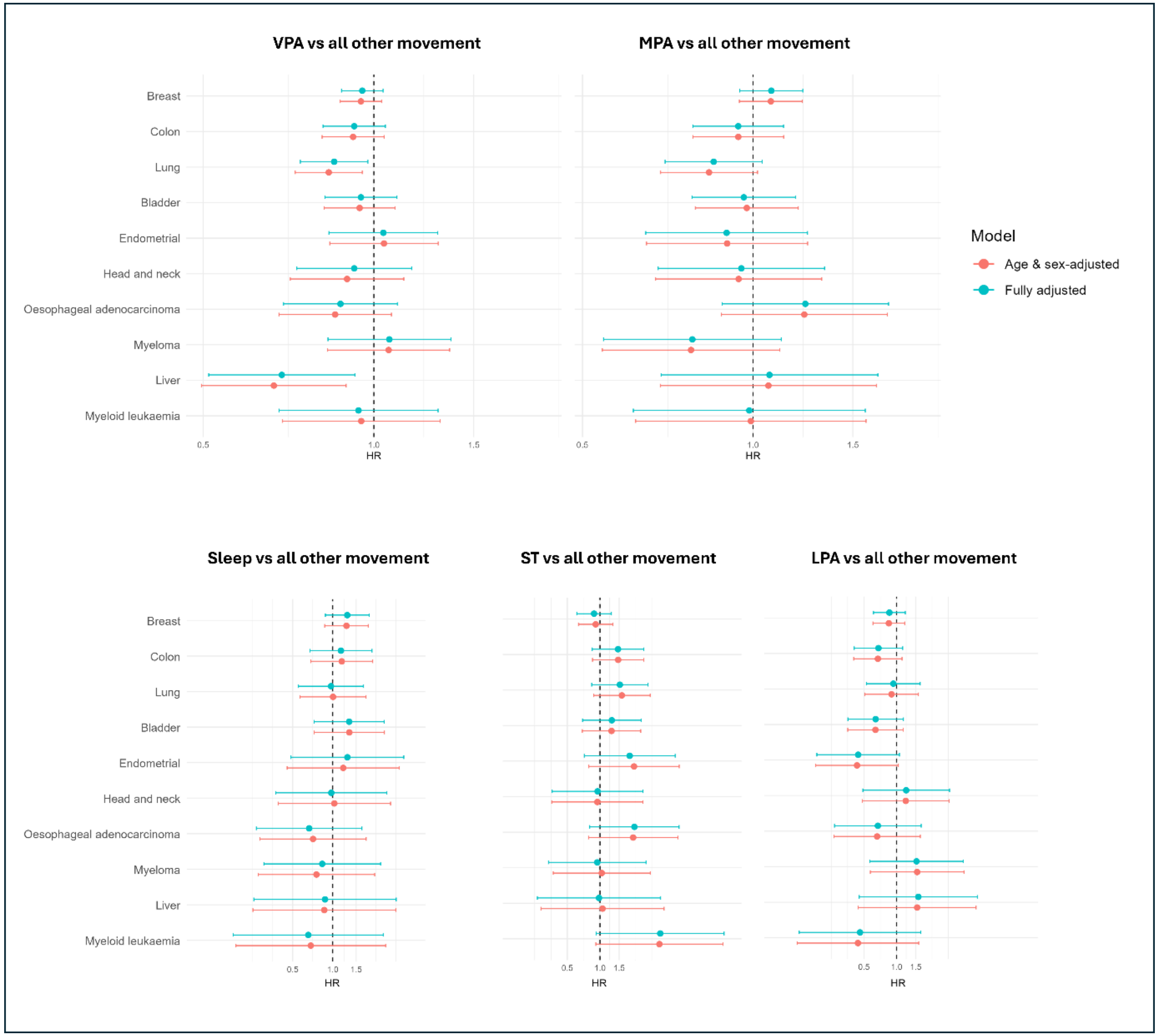

Table S19. Included and excluded participant characteristics at baseline.

		Included Sample		Excluded Sample		p-value
		n=59,218		n=22,666		
**Age (years)**	Mean (SD)	61.7	(7.8)	62.9	(7.8)	**<0.001**
**Sex (female)**	n, %	32,452	55%	13,549	60%	**<0.001**
**Ethnicity (White)**	n, %	56,988	96%	21,632	95%	**<0.001**
**Education**	n, %					**0.007**
College/University degree		25,737	43%	9,997	44%	
A/AS-level		7,966	13%	2,943	13%	
NVQ/HND/HNC		3,235	6%	1,151	5%	
CSE		2,335	4%	832	4%	
O-levels		12,116	21%	4,622	20%	
Other		7,829	13%	3,121	14%	
**Smoking history**	n, %					**<0.001**
Current		3,925	7%	1,633	7%	
Never		34,092	56%	12,621	56%	
Ex		21,201	37%	8,412	37%	
**Medication Use** (Cholesterol lowering, Blood pressure-lowering, Insulin)	n, %	13,549	23%	5,687	25%	**<0.001**
**Alcohol**	n, %					**<0.001**
Above guidelines		22,409	38%	8,423	37%	
Ex		1,545	3%	627	3%	
Within guidelines		33,647	57%	12,944	57%	
Never		1,617	3%	672	3%	
**Family history of Cancer**		15,353	26%	6,424	29%	**<0.001**
**Diet Quality (Fruit/Vegetable consumption) index**	Median (Q1,Q3)	7	(5-10)	7	(5-10)	**0.1953**
**Previous history of CVD**		5,366	9%	2,372	10%	**<0.001**
**PA-related Cancer Incidence**		2,385	4%	-	-	-
**Follow up in years**	Median (Q1,Q3)	8.0	(7.4-8.5)	-	-	-
**Competing risk**	Median (Q1,Q3)	1,552	3%	-	-	-
**Previous history of Cancer**		-	-	7,633	34%	-

Comparisons of categorical variables with chi-squared tests and continuous variables with Wilcoxon signed-rank tests. Totals in excluded sample may vary due to missingness. (CVD: Cardiovascular disease; PA: Physical activity). Excluded sample were missing Physical activity measures, cancer outcomes and/or covariates.
